# Dissecting splicing decisions and cell-to-cell variability with designed sequence libraries

**DOI:** 10.1038/s41467-019-12642-3

**Published:** 2019-10-08

**Authors:** Martin Mikl, Amit Hamburg, Yitzhak Pilpel, Eran Segal

**Affiliations:** 1 Department of Computer Science and Applied Mathematics, Weizmann Institute of Science, Rehovot, 7610001 Israel; 2 Department of Molecular Cell Biology, Weizmann Institute of Science, Rehovot, 7610001 Israel; 3 Department of Molecular Genetics, Weizmann Institute of Science, Rehovot, 7610001 Israel

**Keywords:** Assay systems, Gene regulation, Alternative splicing, Cellular noise

## Abstract

Most human genes are alternatively spliced, allowing for a large expansion of the proteome. The multitude of regulatory inputs to splicing limits the potential to infer general principles from investigating native sequences. Here, we create a rationally designed library of >32,000 splicing events to dissect the complexity of splicing regulation through systematic sequence alterations. Measuring RNA and protein splice isoforms allows us to investigate both cause and effect of splicing decisions, quantify diverse regulatory inputs and accurately predict (R^2^ = 0.73–0.85) isoform ratios from sequence and secondary structure. By profiling individual cells, we measure the cell-to-cell variability of splicing decisions and show that it can be encoded in the DNA and influenced by regulatory inputs, opening the door for a novel, single-cell perspective on splicing regulation.

## Abstract (web summary)

Alternative splicing is regulated by multiple mechanisms. Here the authors employed designed splice site libraries and massively parallel reporter assays to dissect the regulatory complexity and cell-to-cell variability of splicing decisions and to build accurate predictive models.

## Introduction

An alternative splicing event can be the decision whether an exon is included in the mRNA (“cassette exons”), whether an intron is retained (“retained introns”) or which of two alternative donor or acceptor sites is being used (“tandem splice sites”). The fundamental differences between these types entail peculiarities in the mode of regulation. Splicing has been shown to be influenced by a multitude of factors, ranging from local sequence motifs to epigenetic modifications. RNA binding protein (RBP) binding sites^1–3^, DNA methylation^4^, secondary RNA structure^5^, among others, have been implicated in affecting the splicing decision, which makes disentangling the individual contributions and attributing specific functions to these regulatory mechanisms an extremely complex task when investigating native sequence contexts.

Despite extensive research, our understanding of the rules by which sequence determines splicing decisions is limited and to a large extent qualitative in nature. Approaches up to now have mainly relied on RNA sequencing data to build a computational model for alternative splicing^6,7^ or tested the effect of short randomized sequences or point mutations on a nearby constant splicing event^8–11^. This has led to a model predicting the direction of change in alternative splicing between different tissues^6,7^ or the effect of specific point mutations on splicing^10,12^ or identified k-mers influencing selection of specific splice sites^9,10^. However, a comprehensive approach elucidating design principles of different types of alternative splicing in a context-independent manner, utilizing the power of a massively parallel reporter assay, while maintaining expression from a native locus, is still missing. Moreover, investigating splicing regulation is typically based on analyzing relative RNA isoform abundances, disregarding the downstream consequences splicing decisions can have on expression of the corresponding protein isoforms as well as their cell-to-cell variability.

Here we use a combined experimental and computational approach to unravel principles of alternative splicing in a comprehensive and quantitative way. Our approach tests rationally designed sequences and controls the genomic environment by site-specific integration, thereby reducing the regulatory complexity and enabling us to pinpoint causative sequence changes. We construct libraries of altogether 32,789 splice site sequences and measure the effect of targeted sequence manipulations on the ratio between splice isoforms, enabling us to address many of the gaps that exist in our understanding of splicing regulation and elucidate regulatory design principles of splice sites. We follow splicing decisions in individual cells until the final gene product, allowing for a comprehensive view of splicing regulation in light of its downstream consequences and a systematic investigation of cell-to-cell variability in alternative splicing that will help to decipher the rules shaping noise in splicing decisions and its functional implications.

## Results

### High-throughput testing of rationally designed splice sites

**Fig. 1:**
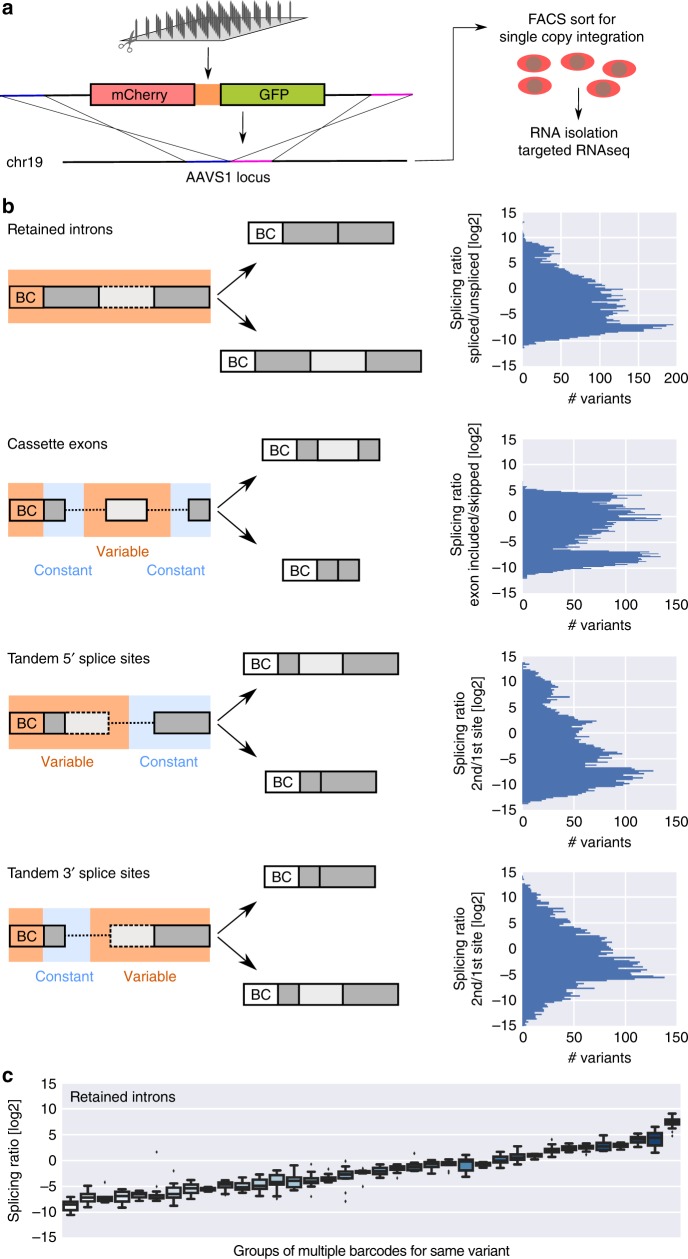
High-throughput testing of rationally designed splice site variants. **a** Outline of the experimental pipeline. Designed synthetic oligonucleotide libraries were cloned in a splicing reporter and integrated in the AAVS1 locus of K562 cells, with a single library variant per cell. After FACS sorting, RNA was isolated and the reporter RNA was sequenced, leading to determination of splicing ratios for each variant. **b** Structure of the variable (orange) and constant (light blue) regions in the four splicing type libraries, with the two splicing outcomes and the corresponding histograms. Splicing ratios close to the upper or lower limit represent predominance of the upper or lower isoform in the schematic, respectively (BC: barcode). **c** Barcode controls for retained intron splicing ratios, box plots for groups of multiple barcodes for the same sequence variant (*n* > 7 for all groups), plotted according to their mean splicing ratio (spliced/unspliced [log2]); boxes show the quartiles of the dataset, whiskers show the range of the distribution not including outliers (displayed as points)

We designed four synthetic libraries of 8551, 9608, 7473, and 7157 oligonucleotides, comprising library-specific common primers, a unique barcode and a 147–162 nt long variable region (Supplementary Fig. 1A). The variable region either contained (a) a retained intron flanked by exonic sequences, (b) a cassette exon flanked by intronic sequences, (c) two alternative tandem 5′ splice sites or (d) two alternative tandem 3′ splice sites. Sets of 38, 134, 81, and 96 native splice site contexts spanning a wide range of splicing ratios (Supplementary Fig. 1B, Supplementary Data 1-4) were used as basis for systematic sequence manipulations. We cloned the synthesized libraries (Agilent) between mCherry and GFP—with or without additional constant regions, depending on the splicing type—and introduced this construct in the AAVS1 locus in the human K562 cell line using zinc finger nucleases, such that every cell has one splicing reporter construct and all the variants have the same genomic environment (see Methods, Fig. 1a, b, Supplementary Fig. 1A). We sorted the mCherry-positive population corresponding to a single integration of the reporter transgene using flow cytometry and collected cells for RNA isolation followed by targeted RNA sequencing to quantify the abundance of different splice isoforms. We report the splicing outcome as the log ratio between the two isoforms (Fig. 1b), i.e., spliced vs. unspliced for retained introns, included vs. excluded for cassette exons, second vs. first splice site for tandem 5′ and 3′ splice sites (Supplementary Data 5-8), as this provides a meaningful measure across all splicing types assayed here and results in a large dynamic range for quantifying effects on isoform ratio.

We confirmed the low technical noise of our system by comparing replicates (Supplementary Fig. 1A) and by examining groups of at least eight independent variants with identical sequences except for the DNA barcode (Fig. 1c, Supplementary Fig. 1C), showing that we can quantify the effect of sequence variations on splicing over a wide dynamic range.

### Tandem donor sites follow first come-first served principle

**Fig. 2:**
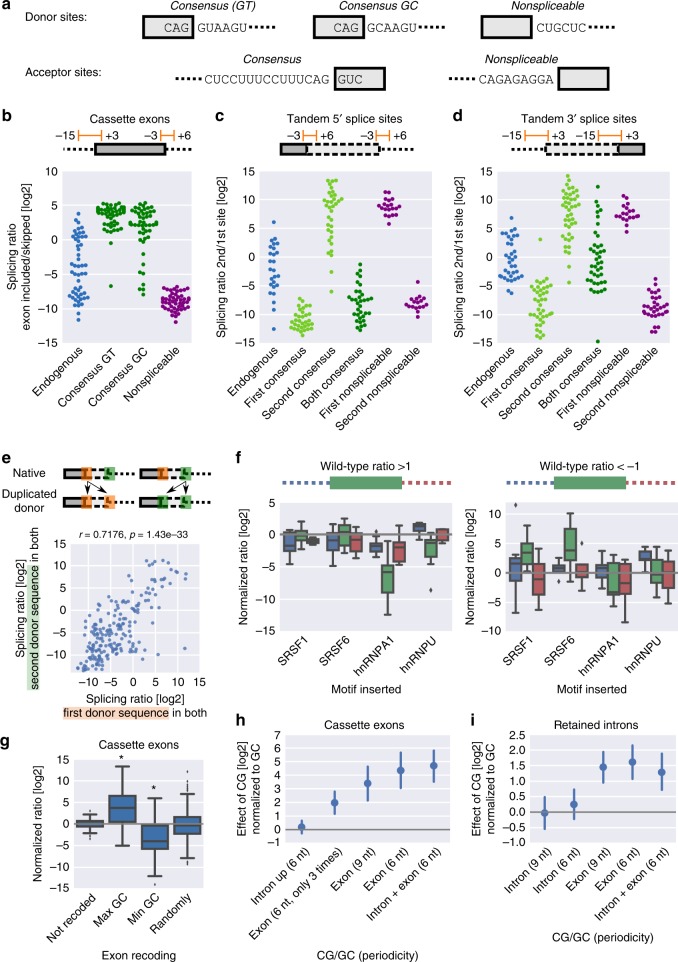
Splice site choice can be efficiently biased by minimal sequence changes. **a**–**d** The indicated splice site mutations (**a**) were introduced at donor (−3 to +6) and acceptor (−15 to +3) splice sites of cassette exons (**b**) or, in a combinatorial fashion, at one or both of tandem 5′ (**c**) or 3′ (**d**) splice sites (Methods). Data points denote splicing ratios of individual variants within the indicated group (*n* = 17–107 for individual groups). Shades of green indicate consensus splice sites, purple indicates nonspliceable sequences. **e** Splicing ratio (2nd/1st) of tandem 5′ splice site variants in which the first splice site sequence was copied to both (orange box), plotted against the splicing ratio of variants from the same context with the second splice site sequence copied to both (green box); each data point constitutes one endogenous context with duplicated splice site sequences of varying length (*n* = 87); Pearson correlation coefficient and the associated *p*-value are stated. **f** Distributions of the effect on the endogenous splicing ratio (= normalized ratio) of introducing a motif for the indicated splicing factor within a given region (blue: upstream intron, green: cassette exon, red: downstream intron) for contexts with a tendency for exon inclusion (left, wild-type splicing ratio >1) or skipping (right, wild-type splicing ratio <−1). Several points of insertion within this region are treated as one set (*n* = 9–20 for each set); boxes show the quartiles of the dataset, whiskers show the range of the distribution not including outliers (displayed as points). **g** Distribution of normalized ratios (to the respective wild-type control) for recoding of cassette exons (*n* = 99, 83, 89, and 292 variants); boxes show the quartiles of the dataset, whiskers show the range of the distribution not including outliers (displayed as points); asterisks indicate statistically significant effects (*p* < 0.05) as determined using Wilcoxon signed-rank test. **h**, **i** Mean and 95% CI of normalized ratios (to the respective wild-type control) for cassette exons (**h**) and retained introns (**i**) in which CG or GC was introduced at the indicated frequency either in the exonic or intronic regions or both (*n* = 17–24 and *n* = 40–58 variants in each group)

To determine how efficiently we can bias splicing across diverse sequence contexts by manipulating only the immediate splice site sequences, we replaced the region around dozens of endogenous splice junctions (−3 to +6 nucleotides for donor and –15 to +3 nucleotides for acceptor sites) with consensus splice sites or nonspliceable sequences (Fig. 2a). Consensus splice sites led to efficient exon inclusion, irrespective of the sequence context (Fig. 2b), with intron-initial GT being more effective than GC (*p* = 1.5 × 10^−4^, Wilcoxon signed-rank test). Introducing an optimal branch point sequence led to a moderate increase in splice site usage in retained introns and cassette exons (Supplementary Fig. 2A, B), but did not generally affect the choice between tandem acceptor sites (Supplementary Fig. 2C). In contexts containing tandem 5′ or 3′ splice sites a consensus sequence at the first or second site led to the expected decrease and increase in splicing ratios (2nd/1st site), respectively (Fig. 2c, d). When both 5′ splice sites were replaced with the consensus sequence, the first one was predominantly used across contexts (Fig. 2c; *p* = 3.3 × 10^−5^, Wilcoxon signed-rank test), resolving earlier conflicting evidence^10,13^. For tandem 3′ splice sites no such preference could be observed (Fig. 2d), indicating that the order on the transcript is a decisive factor in the choice between two donors, but not between two acceptor splice sites. This pattern held true across all potential splice site sequences; assessing the relationship between the difference in splice site strength and the splicing ratio for all library variants showed that for equal strength the first splice site is favored for tandem 5′, but not 3′ splice sites (Supplementary Fig. 2D). The sigmoid shape of the relationship suggests that when splice site strengths are different the stronger one dominates. Artificially creating a situation of equal splice site strength by copying the first (orange) splice site sequence to the second donor showed high correlation (Fig. 2e) with the inverse configuration (second (green) splice site sequence copied to the first donor), suggesting that the immediate splice site sequence and the larger sequence context affect splicing independently from one another. Situations where the first come-first served rule is broken are therefore mostly set by sequence properties away from the immediate splice site.

To quantify the effect of additional regulatory elements, we introduced binding sites for common splicing factors and splicing-regulatory sequence motifs from previous studies^9,10^ at different positions in dozens of native contexts and tested for their influence on donor and acceptor sites. Motifs often showed location- and splicing type-dependent activity (Supplementary Fig. 2E). Single binding sites could have dramatic effects on exon inclusion levels (Fig. 2f). Their activity often depended on splice site strength (e.g., hnRNPA1, SRSF6), but less on the precise binding site location (Supplementary Fig. 2F), and was affected by co-insertion of other binding sites (e.g., hnRNPA1 + SRSF1; Supplementary Fig. 2G).

### GC content and CG dinucleotides affect splicing decisions

Introns and exons differ in their GC content^14^. To assess the potential for regulation based on GC content alone, given a desired protein outcome, we recoded native splice site regions and measured the effect on splicing. Recoding of a cassette exon for highest or lowest possible GC content had strong opposite effects of similar magnitude (mean fold change ~2^4^, Fig. 2g), indicating that endogenous cassette exons tend to be not committed to either high or low splicing efficiency based on their GC content alone, leaving a lot of regulatory potential to influence splicing in either direction.

DNA methylation at the cysteine residue in CG dinucleotides has been proposed as another means for regulating splicing^4^. We introduced CG or GC at different frequencies in 30 cassette exons and quantified the effect conferred by introduction of CG, which is potentially methylated, as opposed to GC (Fig. 2h). When introduced in the exon, CG biased splicing towards inclusion of the exon (*p* = 1.0 × 10^−22^, Wilcoxon signed-rank test) in a dose-dependent manner. Having additional CGs in the intron did not interfere with this positive effect (*p* = 0.22), suggesting that the presence of CGs increases usage of an already defined cassette exon, as opposed to making it distinguishable from the surrounding intronic region. A differential effect of CG vs. GC dinucleotides could also be observed for exons surrounding retained introns (Fig. 2h; *p* = 9.0 × 10^−11^, Wilcoxon signed-rank test), supporting recent observations showing a relationship between loss of DNA methylation and increased intron retention^15,16^.

### Coordination and antagonism shape splicing decisions

**Fig. 3:**
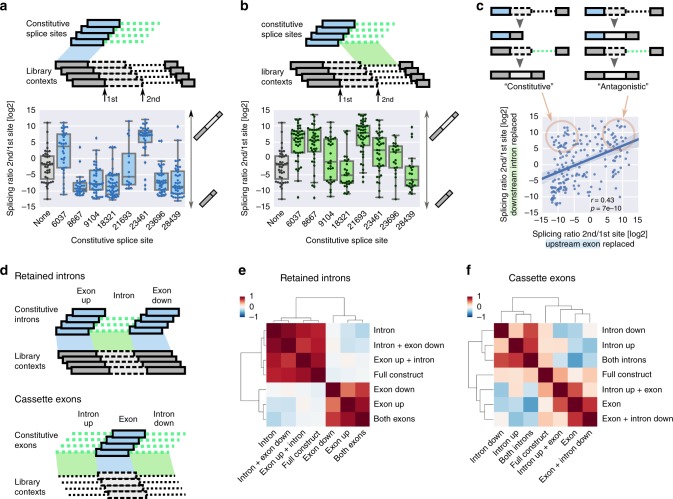
Antagonistic interactions between building blocks shape the overall splicing outcome. **a**, **b** Distribution of splicing ratios (2nd/1st) in tandem 5′ splice sites in variants with the upstream exon (**a**, blue) or the downstream intron (**b**, green) replaced with the corresponding exonic or intronic part from a set of eight constitutive 5′ splice sites (*n* = 17–47 in each group); gray box plots and data points denote wild-type splicing ratios; boxes show the quartiles of the dataset, whiskers show the range of the distribution not including outliers (displayed as points). **c**. Each data point represents the log ratio (2nd/1st splice site) for a pair of exonic (blue, *x*-axis) and intronic (green, *y*-axes) from the same native constitutive donor site placed in a library context (*n* = 279; based on the same data as the box plots in **a**, **b**). The schematic on top illustrates the expected behavior in the case of “constitutive” splicing behavior triggered by the introduced component (left) or antagonistic (right, in the example shown: an intronic component (green) promotes usage of the 2nd (the adjacent) splice site and an exonic component (blue) from the same native donor site is not able to trigger usage of the first (the adjacent) splice site, both leading to high splicing ratios (2nd/1st)). Pearson correlation coefficient and the associated *p*-value are stated. **d** Schematic for the replacement of components of the retained intron (top) and cassette exon (bottom) library contexts with elements from a set of “constitutive” splice site sequences (with no evidence in RNAseq data for retention of the intron or skipping of the exon, respectively. **e**, **f** Clustered heatmaps showing Pearson correlation coefficients for all pairwise combinations of effects on splicing ratio in (**e**) retained introns or (**f**) cassette exons conveyed by the indicated elements across contexts (*n* = 96–215 and 48–143 for each combination)

To assess the potential of individual building blocks to confer regulatory properties from one context to another, we substituted exonic and intronic components of alternative splice sites with sequences from native, constitutive splice sites without evidence for alternative splicing (Fig. 3a, b, Supplementary Fig. 3A, B, Supplementary Data 9-10). The naive assumption would be that sequences surrounding constitutively used splice donors promote usage of the adjacent splice site when introduced in our library contexts, i.e., splicing at the first donor (= low splicing ratio 2nd/1st) when the upstream exon was replaced with the 3′ end of constitutive exons or splicing at the second donor ( = high splicing ratio 2nd/1st) when the downstream intron was replaced with the 5′ end of constitutive introns. Both the preceding exon and the downstream intron itself can indeed promote splice site usage (low splicing ratios in Fig. 3a, high splicing ratios in Fig. 3b, with exons showing a slightly stronger tendency to promote usage of the adjacent splice site, *p* = 0.004, Wilcoxon signed-rank test). However, despite coming from “constitutively” used splice sites, not all sequences triggered usage of the adjacent 5′ splice site (Fig. 3a, b).

We then compared the effect of exon-intron pairs from constitutive splice sites on the splicing behavior of our library contexts (Fig. 3c). If both the exon and the intron trigger constitutive usage of the adjacent splice site (as in the case of constitutive splice site 8667; Fig. 3a, b), this would result in data points lying in the upper left corner of Fig. 3c. However, when analyzing all sequence combinations tested, effects for pairs of exon and intron sequences taken from the same native, constitutive context showed positive correlation, with values spanning the full range of splicing ratios (Fig. 3c), indicating antagonistic behavior of exonic and intronic components of endogenous splice sites. This suggests a design principle of endogenous donor sites in which only one of the components, either the exon or the intron, is a strong promoter of splicing. In the endogenous context (and in the absence of a competing donor site) this is enough to ensure efficient splice site usage. This design principle thus avoids redundancy and reduces the constraints on the DNA sequence, but could also be a mechanism to allow for evolutionary plasticity.

The potential of 3′ ends of constitutive introns to promote splicing can generally be transferred to our library contexts, leading to low splicing ratios (Supplementary Fig. 3A). Exonic and intronic components of splice acceptors did not show the antagonistic behavior observed for components of donor sites (Supplementary Fig. 3C), largely due to the weak effect of the downstream exon on splicing behavior (Supplementary Fig. 3B), which leaves the regulatory burden on the intronic part.

To test for coordinated effects between the building blocks of endogenous splice sites, we replaced exonic and intronic components of 38 retained introns and 30 cassette exons from our library contexts with sequences from 5 to 6 length-matched constitutive introns (and their surrounding exons) and constitutive exons (and their surrounding introns) without any evidence for intron retention or exon skipping in RNAseq data (Supplementary Data 11–12), respectively (Fig. 3d). Like in the case of donor splice sites, testing the entire constitutive splice sites with the flanking sequences (‘full construct’) in the context of our reporters did not generally lead to maximal levels of exon inclusion/intron removal (Supplementary Fig. 3F, H), corroborating a view according to which there is no dichotomy of constitutive vs. alternative splice sites.

For sequence elements that contain each other (i.e., intron vs. intron + exon down) high correlation in associated splicing ratios could be observed, as well as in the case of the exon upstream and the exon downstream of retained introns (Pearson *r* = 0.54, *p* = 6 × 10^−11^, Fig. 3e, Supplementary Fig. 3D), suggesting coordination in their effect on splicing efficiency of the intron in between in the native context. In the case of cassette exons, intronic and exonic elements showed strong negative correlation (*r* = −0.6, *p* = 4 × 10^−7^, Fig. 3f, Supplementary Fig. 3E), arguing for antagonistic effects creating a balance between components favoring or disfavoring splicing that gives rise to the endogenous splicing decision. This was not due to an underlying difference in the distribution of splicing ratios between the groups: Both exonic and intronic components of cassette exons (but not retained introns; Supplementary Fig. 3F–I) had similar potential to promote splicing, with no significant differences between the relevant groups (Supplementary Fig. 3H, I, exon vs. both introns, *p* = 0.34, Mann–Whitney U test). Antagonism between exons and introns has been reported for individual splicing factor binding sites^17^, and here we show that this also holds true for exons and their surrounding introns as a whole.

### Prediction of splicing ratios from sequence and structure

**Fig. 4:**
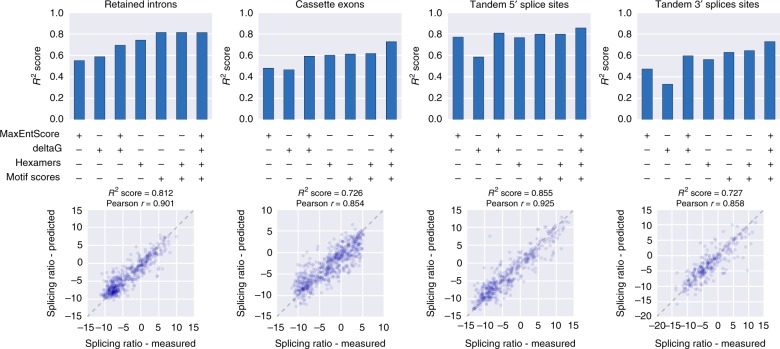
Accurate prediction of splicing ratios. *R*^2^ scores for predicting splicing ratios of a set of variants not used at any point to build the model (10% of library variants) by Gradient Boosting Regression with the indicated sets of features (top) and measured splicing ratios vs. predictions based on a combination of hexamer counts, RBP motif scores, MaxEntScan scores and secondary structure features (bottom) for the indicated splicing types

Having measurements for large collections of splice site variants from a constant genomic environment, we wanted to undertake a task that has proven challenging in the field of splicing: To quantitatively predict splicing based on sequence. We used splice site strength (as determined using MaxEntScan^18^), hexamer counts, cumulative binding scores for 160 RNA binding proteins (ATtRACT^19^) and RNA secondary structure, alone and in combination, as features and trained a Gradient Boosting Regressor (Methods; Fig. 4, Supplementary Fig. 4A).

Using only the predicted minimum free energy (ViennaRNA^20^) of regions around splice sites, we achieved *R*^2^ scores between 0.33 and 0.59 (Fig. 4), indicating that secondary structure alone, without any sequence information, can be predictive of splicing outcome. This is in line with the strong effect introducing a hairpin around or downstream of splice sites has on splicing (Supplementary Fig. 4B) and the pronounced preferences for secondary structure (Supplementary Fig. 4C), e.g., pairing at the pyrimidine tract being associated with more efficient splicing of (potentially retained) introns. This observation is in contrast to the expectation that an accessible poly-pyrimidine tract would be beneficial for efficient splice site usage and might reflect a strategy found in yeast, where secondary structure before the acceptor site helps splicing by reducing the distance between a distant branch point and the splice site^21,22^. Tandem 3′ splice sites on the other hand showed a preference for the area of the pyrimidine tract to be unpaired (Supplementary Fig. 4C), suggesting different structural properties of a splice site being important depending on the context, i.e., if it has to compete with a neighboring splice site or to recruit the splicing machinery to a weak intron.

In the case of tandem 5′ splice sites, the two donor sites show differences in their importance and effect on the prediction, especially in the case of secondary structure (Supplementary Fig. 4D), recapitulating the dominance of the first splice site identified by testing rationally designed variants (Fig. 2c).

Taking a naive approach and using counts of all possible hexamers in the regions surrounding the splice sites (Supplementary Fig. 4A) allowed prediction of splicing ratios of unseen variants with higher accuracy (*R*^2^ scores between 0.56 and 0.76) and recovered known 5′ and 3′ splice site sequences in an unbiased way (Supplementary Fig. 4E). The most important features between splicing types were the canonical donor and acceptor splice site sequences as well as pyrimidine-rich features in a position-dependent manner (e.g., GTAAGT in tandem 5′ splice sites, Supplementary Fig. 4E).

As some of the hexamers used for the prediction might represent binding sites for splicing factors, we used the cumulative binding scores of each motif in a database of 1174 motifs for 160 RNA binding proteins (ATtRACT^19^) for exonic and intronic regions in our variants as features for our model and let the algorithm select the relevant ones (Supplementary Fig. 4A, Methods), further increasing prediction scores (Fig. 4).

Building models based on all our feature sets allowed us to quantitatively predict splicing log ratios of unseen variants for all splicing types with high accuracy (*R*^2^ scores between 0.726 and 0.855). Many potential splicing factor binding sites were important for the prediction (Supplementary Fig. 5) and affected splicing ratio predictions consistent with their reported function (e.g., members of the SR protein family of splicing factors generally considered to promote exon inclusion^1,23^), although some of the effects might be due to other sequence properties of the motifs (e.g., GC content).

To test if a model trained on our data is able to predict splicing behavior also in unrelated datasets we tested it on other MPRAs (MaPSy^24^ and Vex-seq^8^ for cassette exons and Rosenberg et al.^10^ for tandem 5′ splice sites). Both MaPSy and Vex-seq are designed to screen for effects of genetic variation on splicing and Rosenberg et al.^10^ are testing the influence of 25 nt regions on nearby (constant) competing 5′ splice sites. These studies therefore constitute conceptually very different approaches with a different underlying study design. Nevertheless, a model trained on our data could predict splicing behavior of variants from these MPRAs reasonably well (Pearson *r* between 0.33 and 0.58, Supplementary Fig. 6A). To predict the effect of sequence variation we calculated the paired difference between the splicing ratios predicted for wild type and mutant. Although our model was not optimized and trained for prediction of single nucleotide variant effects, we achieved prediction scores comparable to state-of-the-art predictors (Supplementary Fig. 6B, C, Pearson *r* = 0.31 and 0.3 on MaPSy and Vex-seq data, respectively, as compared to Pearson *r* values of 0.37 and 0.26–0.68, respectively, for a set of predictors recently tested on the same datasets^25^). Similar (Pearson *r* = 0.32; MaPSy, Supplementary Fig. 6B) or worse (Pearson *r* = −0.02; Vex-seq, Supplementary Fig. 6C) performance was observed when using a part of the MaPSy and Vex-seq data, respectively, as training set and scoring performance on the rest, showing that the performance of our model relies on the complexity and diversity of the training data.

### Differential downstream fates linked to splicing decisions

**Fig. 5:**
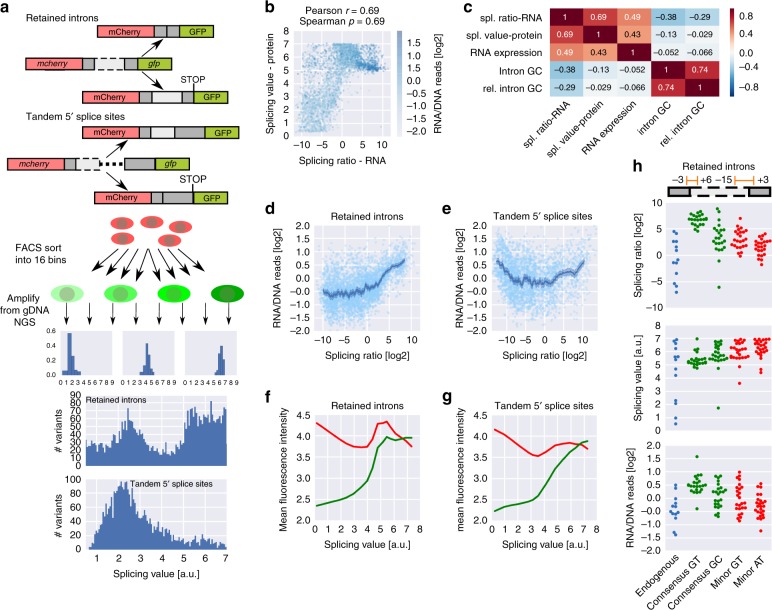
Quantifying protein isoform ratios reveals differential posttranscriptional fates. **a** Outline of the experimental pipeline for obtaining protein-based splicing measurements for retained introns and tandem 5′ splice sites. **b** RNA-based splicing ratios plotted against protein-based splicing values for the retained intron library; the color intensity denotes the RNA expression levels (dark blue corresponds to high and light blue to low RNA expression levels (log2(RNA/DNA reads)). **c** Pearson correlation coefficients between RNA-based splicing ratios, protein-based splicing values, RNA expression levels (log ratio of RNA/DNA reads), intronic GC content and relative intronic GC content (normalized to the GC content of the surrounding exons). **d**, **e** Log ratios of RNA/DNA reads (= RNA expression levels) plotted against splicing ratios for the retained intron (**d**) and tandem 5′ splice sites (**e**) library. **f**, **g** Mean mCherry (red) and GFP (green) fluorescence intensity for cells from the retained intron (**f**) or tandem 5′ splice sites library (**g**) sorted into each of the 16 bins are plotted against the respective splicing value (i.e., the median log ratio of GFP/mCherry fluorescence intensity). **h** Data points denote the RNA-based splicing ratios (top), protein-based splicing values (middle) and log ratios of RNA/DNA reads (bottom) of individual variants with the indicated sequence (endogenous or a consensus sequence) at donor and acceptor splice sites (*n* = 22–59); green indicates processing by the major and red processing by the minor spliceosome

To be able to follow splicing decisions in individual cells to the final gene product, we constructed our library in a way that allows us to quantify splicing with a bifluorescent reporter^26^ in large scale (Fig. 5a). In the case of retained introns, only if the intron is removed is the downstream *gfp* in frame and are both mCherry and GFP made into protein. In the case of tandem 5′ splice sites, GFP expression is dependent on usage of the second donor site; usage of the first donor site leads to expression of mCherry alone. The ratio of GFP vs. mCherry fluorescence is a sensitive measure of protein isoform ratios in individual cells.

We sorted the pool of cells, each carrying one variant, into 16 bins according to their GFP/mCherry ratio and sequenced genomic DNA from all the bins to unravel the distribution of each variant (Fig. 5a), which provides a measure of both the population average as well as the variability of splicing decisions at the single-cell level.

We previously demonstrated that similar approaches are highly accurate and reproducible^27,28^. Results for groups of identical barcodes (Supplementary Fig. 7A) and the associated bin profiles (Supplementary Fig. 7B) corroborate the low technical noise we are able to achieve.

RNA- and protein-based readouts for retained introns are well correlated (Pearson *r* = 0.69; Fig. 5b, c; Pearson *r* = 0.51 for tandem 5′ splice sites; Supplementary Fig. 7C), but show particular differences. RNA expression levels increase with efficiency of intron removal (Fig. 5c, d), but protein expression levels are equally high at low and high splicing values (Fig. 5f), suggesting that—at least in this context of a single-intron gene—transcript variants lacking clear splicing signals can yield similar translational outputs as efficiently spliced variants. This increased translational output at low splicing levels might be due to the transcript not being recognized by the splicing machinery at all (and potentially retained in the nucleus). No such discrepancy could be observed for alternative 5′ splice sites (Fig. 5e, g), where the decision is not whether to splice or not, but which splice site to use. Variants with intermediate splicing levels are more likely to be degraded due to failed processing, resulting in both lower RNA and protein levels (Fig. 5e, g). A similar effect can be observed for retained introns with intermediate splicing ratios at the protein level (Fig. 5f).

Relative intronic GC content is negatively correlated with the RNA, but not the protein splicing ratio (Fig. 5c), indicating that influences of GC content on splicing efficiency are buffered at the protein level, possibly through a negative effect of high intronic GC content on nuclear export or translation of the unspliced isoform. This hypothesis is corroborated by testing library variants in which the exonic and/or intronic components have been recoded to have different GC contents: Recoding the intron for maximal GC content led to lower ratios of spliced/unspliced transcripts, but did not significantly affect protein-based splicing values (Supplementary Fig. 7D). To identify specific sequence features mediating the discrepancy between RNA and protein we computed the difference in Pearson correlation coefficients between all intronic hexamer counts and either RNA- or protein-based splicing readouts (Supplementary Fig. 7E). Several properties of optimal introns (low GC content (Supplementary Fig. 7F; see also above), the consensus 5′ splice site GTAAGT and pyrimidine-rich features (Supplementary Fig. 7E)) were among the hexamers with the largest difference between RNA isoform ratios and protein-based splicing values (Supplementary Fig. 7G).

We therefore compared 38 native retained introns in which the immediate splice sites were replaced with either the consensus sequences for the major spliceosome or less efficiently processed alternatives (5′-GC, minor spliceosome). While a consensus for the major spliceosome resulted in higher ratios of spliced/unspliced transcripts (Fig. 5h, top), corresponding variants with a 5′-GC or minor spliceosome-specific sequences were associated with a higher fraction of the spliced, GFP-containing isoform on the protein level (Fig. 5h, middle).

Mean GFP intensity reaches a plateau around splicing values corresponding to processing of consensus sites by the major spliceosome (splicing value ~5; Fig. 5f). Higher GFP/mCherry ratios appear to be due to lower expression levels of the mCherry-only protein product, indicating that the observed discrepancy is due to reduced translational output from the unspliced isoform. RNA expression levels are strongly influenced by the efficiency of splicing and the machinery involved. GT-consensus sequences lead to significantly increased RNA levels (Fig. 5h, bottom, *p* = 0.0026, Wilcoxon signed-rank test), likely exceeding the capacity of downstream processing steps.

Our observations might reflect a bottleneck due to inefficient processing. Here, a larger proportion of RNA molecules than in the case of the optimal consensus would not be processed immediately after transcription. This unspliced pre-mRNA is not exported and translated, but is detectable on the RNA level. Our results therefore provide evidence that less efficient splicing can yield more clear-cut choices between protein isoforms than maximal splicing efficiency.

### Splicing noise can be affected by regulatory inputs

**Fig. 6:**
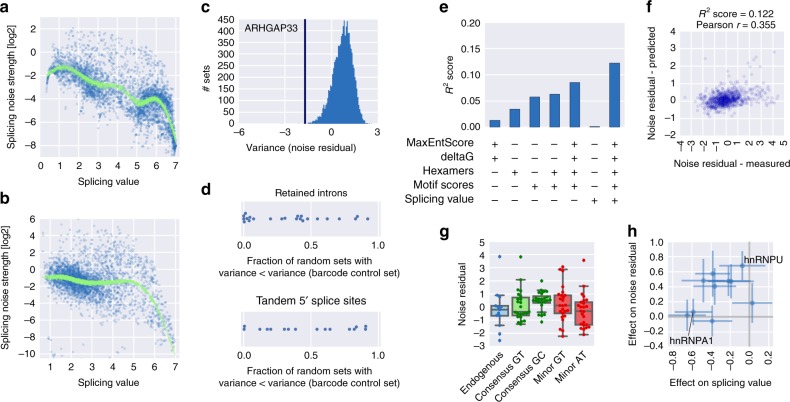
Cell-to-cell variability of alternative splicing can be encoded in the DNA sequence. **a**, **b** Noise strength (variance/mean) is plotted against mean splicing values for the retained intron (**a**) and tandem 5′ splice site (**b**) libraries. **c** Example result for comparing the variance of noise residuals for groups of individual barcode controls to variances for 10,000 sets of equal size, randomly picked from library variants in the same range of splicing values. The vertical blue line indicates the variance of the barcode control group. **d** The *x*-axis denotes the fraction of random sets with a variance smaller than the one of the corresponding barcode control set for groups of identical splice site sequences from the retained intron (top) and tandem 5′ splice site (bottom) libraries. **e** *R*^2^ scores for predicting splicing noise residual using the indicated features. **f** Measured noise residuals are plotted against predictions based on the full set of features in panel (**e**). **g** Distribution of noise residuals in retained intron library variants with the indicated sequence (endogenous or a consensus sequence) at donor and acceptor splice sites; boxes show the quartiles of the dataset, whiskers show the range of the distribution not including outliers (displayed as points); green indicates processing by the major and red processing by the minor spliceosome. **h** Mean and standard error of the mean of the effect on splicing values (*x*-axis) and on noise residuals (*y*-axis) of introducing a motif for the indicated splicing factor in and around retained introns. Several points of insertion are treated as one set (*n* = 4–18 in each group)

Evidence from transcriptome-wide approaches^29,30^ and studies focusing on individual genes^26,29^ provide examples for cases where bulk splicing measurements do not adequately reflect splicing decisions on the single-cell level, with potentially far-reaching functional biological implications as observed in other areas of gene regulation^31^. Our approach allows us to quantify the variability in splicing between cells based on the distribution across bins (Supplementary Fig. 8A). The strength of splicing noise (variance/mean) is negatively correlated with splicing efficiency (Fig. 6a). As the relationship between splicing noise strength and mean splicing value appears to be non-linear and to account for the dependency on expression levels (RNA/DNA reads, Supplementary Fig. 8B, C), we fitted a generalized additive model (see Methods) and used the deviation from this fit as a noise measure and refer to it as the noise residual.

But can the cell-to-cell variability of splicing be determined by the DNA sequence? To test this, we used sets of at least eight identical splice site sequences and checked if the variability within these sets is smaller than would be expected by chance. For each set, we compared the variance of noise residuals to the distribution of variances from 10,000 randomly chosen sets of splicing value-matched variants (Fig. 6c). While some sequences containing a retained intron show significantly lower variance in noise levels, for other retained introns and for tandem 5′ splice sites the within-group variability is as high as in randomly picked sets (Fig. 6d), indicating that noise level can be encoded in the sequence, but this is not implemented for every splicing event and splicing type.

Despite this limitation we could account for 8.5% of the variability with a predictive model using our set of sequence and structural features (Fig. 6e). As we corrected for the association between noise and mean splicing value, the latter by itself was not predictive of the noise residual. Interestingly, adding the mean splicing value to the full set of features further increased the performance of prediction to *R*^2^ = 0.122 (Fig. 6e, f), pointing at interactions between mean splicing value and other features.

Can splicing noise be affected by splice site properties and regulatory inputs? Replacing the region around the endogenous splice sites (−3 to +6 nucleotides for the donor and –15 to +3 nucleotides for the acceptor) with consensus splice sites with an intron-initial GC led to a significant increase in noise residual compared to the corresponding endogenous sequences (Fig. 6g, *p* = 0.028, Wilcoxon signed-rank test). This might be a consequence of the greater uncertainty in the recognition of GC-intial introns due to the mismatch in base pairing with the U1 snRNA. As expected given the stochastic nature of noise properties of tandem 5′ splice sites (Fig. 6d, bottom), no changes in noise residual could be observed after introducing consensus or nonspliceable donor sequences (Supplementary Fig. 8D). Introduction of splicing factor binding sites in and around retained introns showed a tendency to lower mean splicing values and increase noise residuals (Fig. 6h), while some splicing factors showed decoupling between the two effects (e.g., hnRNPU and hnRNPA1, Fig. 6h), with location-specific effects, e.g., for hnRNPU (Supplementary Fig. 8E).

## Discussion

Here, we used rationally designed libraries, consisting of altogether 32,789 variants, to address fundamental questions in splicing regulation. This allowed us to dissect and compare the different regulatory inputs in a quantitative way and identify design principles of alternative splicing events, considering the process in its entirety, from the processing of the RNA to the level of the final functional gene product. Our study goes beyond previous approaches by (a) yielding readouts for RNA and protein isoform ratios and expression levels, (b) making use of a fully designed sequence library, allowing us to reduce the complexity of splicing regulation, (c) integrating each variant in the same genomic location, thereby mimicking expression from a wild-type locus, (d) surveying different splicing types in a comprehensive and comparative way, (e) testing our targeted sequence manipulations in dozens of contexts, eliminating potential biases due to specific effects of sequence changes on the one splicing event typically used in a reporter assay. The importance of incorporating this context-dependence is underscored by a recent study showing that even the immediate donor splice site sequence exhibits context-specific preferences^11^.

Using this approach, we can reproducibly detect even small changes in splicing ratios and quantitatively predict splicing of novel variants with high accuracy (*R*^2^ between 0.73 and 0.85). Our approach to elucidating the context-independent principles of splicing regulation is complementary to studies using endogenous RNA sequencing data to establish a splicing code for prediction of drastic changes in splicing behavior between cell types^6,7^. Our model can be applied to other datasets, but due to differences in the experimental layout and the sequence context the accuracy is lower (Pearson *r* between 0.34 and 0.58 for HAL, MaPSy, and Vex-seq data), attesting to the important contribution of additional factors on splicing behavior. Many other predictors focus on variant effects. Although our model was built to predict splicing behavior of a sequence as a whole and not the effect of single nucleotide changes and has not been trained on appropriate data, it is still able to predict the effect of DNA variations reasonably well (Pearson *r* between 0.29 and 0.31 for Rosenberg et al.^10^, MaPSy^24^ and Vex-seq^8^ data), but does not outcompete dedicated complex models like MMSplice^25^.

Our results show that it is relatively straightforward to build an optimal splice site; simply using the consensus splice site sequence can efficiently trigger splicing, no matter what the surrounding sequences. Large effect sizes can be achieved with even single splicing factor binding sites, altering codon usage and introducing CG dinucleotides, demonstrating that each regulatory input by itself has the ability to significantly bias splicing in most native contexts. And yet, cells evolved to have seemingly suboptimal splice sites, which maximizes the potential for dynamic regulation, but can also serve to ensure optimality at the level of protein isoforms.

Splicing occurs at the RNA level, but it is typically the resulting protein products whose functional differences constitute the significance of this process. Our dual RNA- and protein-based assay revealed properties of splice sites associated with differential downstream fates of isoforms and highlighted that capturing a snapshot at the RNA level might not always reflect the consequences of an alternative splicing event at the level of the final functional gene product.

While the role of noise in other aspects of gene expression has attracted a lot of attention over the last years^32–34^, assessing the variability of splicing decisions has been lagging behind, largely due to technical limitations. Even single-cell RNAseq approaches are limited in their power to detect cell-to-cell differences between splice isoforms differing in only a couple of nucleotides. Here, we established an assay that is able to assess the cell-to-cell variability of splicing decisions in large scale by measuring the protein output of alternative isoforms. We show that the level of stochasticity can be encoded in the DNA. In general, our data present noise as a complex property of splicing events, which is in part a passive consequence of the stochastic nature of gene expression and the uncertainty associated with intermediate splicing efficiencies, but can be influenced by specific sequence elements and properties of a splice site.

## Methods

### Synthetic library—general design notes

Oligonucleotides were designed to maintain a constant length of 210 nt. Restriction sites used for cloning and splice site sequences apart from the assayed donor and acceptor sites were excluded from the design. All the variants were composed of an 18 nt forward primer, 12 nt barcode sequence, 162 nt variable region and 18 nt reverse primer sequences. Barcodes were designed to differ from any other barcode in the library in at least 3 nt. In the case of cassette exons and tandem 3′ splice sites, which required a subsequent cloning step and therefore additional internal restriction sites, SpeI and AatII sites were introduced after the barcode sequence with a 3 nt spacer between them, leaving 147 nt for the variable region. The unique primer sequences at the 5′ and 3′ ends were used for targeted amplification of the variants from the pool of synthesized oligonucleotides.

### Synthetic library—selection of contexts

For all four libraries used here, endogenous sequence contexts (38, 134, 81, and 96 for the retained intron, cassette exon, tandem 5′ and tandem 3′ splice sites libraries, respectively; Supplementary Data 1–4) were selected based on (a) prior testing in a low throughput pilot screen in the same context or (b) by selection of suitable splice sites from publicly available RNA sequencing data for K562 cells (Encode, polyA RNA-seq of K562, Gingeras Lab, accession number ENCFF000HFA; intron length 70–118 nt, exon length 23–89 nt, distance between tandem 5′ splice sites 2–77 nt, distance between tandem 3′ splice sites 2–59 nt). Contexts were chosen such that a wide range of putative splicing ratios would be covered and the alternative splicing event would lead to isoforms with a different downstream reading frame used. In the case of retained introns, a frame shift was introduced in the intron unless intron retention already led to one or the intron contained a stop codon, in order to allow for discrimination of the isoforms on the protein level based on GFP being in frame in the spliced isoform. Retained introns/cassette exons and their flanking regions had to fit into the 162/147 nucleotide long variable region. As alternatively spliced introns and exons tend to be short, this did not constitute a severe limitation for the design.

### Synthetic library—design of individual subsets

For each of the subsets in the libraries, a set of contexts from the previously assembled pool (as described above) was chosen based on the requirements of the specific question to be addressed, e.g., regarding the properties of the intron/alternative exon (length etc.), complexity of the design scheme and required statistical power. Design of subsets was carried out in Python.

Multiple barcode controls: For each library we selected 25–40 splice site sequences expected to span a large range of splicing ratios and generated at least eight variants with identical variable region, but different barcodes.

Splice site mutations (subsets ending in “constvar” in Supplementary Data 5–8): For replacing the immediate splice site sequence with consensus and nonspliceable sequences, the following sequences were introduced: Donor splice sites: consensus (−3:+6)—CAGGTAAGT, nonspliceable (0:+6)—CTGCTC, GC-consensus (−3:+6)—CAGGCAAGT, U12-AT (0:+9)—ATATCCTTT, U12-GT (0: +9)—GTATCCTTT. Acceptor splice sites: consensus (−15:+3)—CTCCTTTCCTTTCAGGTC, U12-acceptor (−19:+3)—TTCCTTAACTTCCTTTCAGATC, branch point (−26:−21)—CTCAC.

Splice site switching/duplication (subsets ending in “switchvar” in Supplementary Data 5–8): For 58 and 43 contexts containing tandem 5′ and 3′ splice sites, the immediate splice site sequences of variable length (9, 6, and 3 nt on the exonic side or increasing sequence portions (in increments of 3 nt) on the intronic side, up to the distance between the two splice sites) from either the first or the second splice site were used to replace the endogenous sequence in the respective other, leading to identical sequences upstream or downstream of the two splice sites.

Splicing factor binding sites (subsets ending in “SFvar”, “SFcombvar” and “SFRosenberg” in Supplementary Data 5–8): Binding sites for SRSF1 (TCACACGAC), SRSF2 (TGGCCTCTG), SRSF5 (TTCACAGGC), SRSF6 (CTGCGTCGA), hnRNPA1 (TTAGGGAAC), hnRNPG (CAAGTGTTC), and hnRNPU (TTGTATTGC), based on reports in the literature and experimental considerations (avoiding stop codons, restriction sites and homopolymers) were introduced at −58:−49, −49:−40, −40:−31, +5:+14, +9:+18 or +14:+23 relative to an acceptor and at −30:−21, −21:−12, −14:−5/−12:−3,+3:+12/+4:+13/+6:+15 or +12:+21/+13:+22/+15:+24 relative to a donor site, depending on the specific requirements imposed by the experimental design. Pairwise combinations of SRSF1, SRSF5, hnRNPA1, and hnRNPU were introduced to assay functional interactions, keeping a minimal distance of 9 nt between binding sites. Sequence motifs identified in previous studies were introduced into the same positions. Specifically, the sequences were CGACGTCGA, CAGAAGAGT, CGAAGATGT, CGCAAGAGT (“enhancers”), CCCAGCAGT, CCTTTTAGT, CCTAGTAGT (“silencers”), CAAAGAGGT, CAAACTTGT, CAACCTTGT (“neutral”), based on Ke et al. (2011) and adapted to accommodate the above mentioned experimental considerations. Hexamers (GENsil (“general silencing”): GTGGGG, E5enh (“enhancer in the alternative exon between tandem 5′ splice site”): CACCGC, E5sil (“silencer in the alternative exon between tandem 5′ splice site”): GGTGGG, I5enh (“enhancer in the intron downstream of tandem 5′ splice sites”): TTGTTC, I5sil (“silencer in the intron downstream of tandem 5′ splice sites”): CGAACC, E3enh (“enhancer in the alternative exon between tandem 3′ splice site”): CGAAGA, E3sil (“silencer in the alternative exon between tandem 3′ splice site”): GGGGGG, I3enh (“enhancer in the intron upstream of tandem 3′ splice sites”): TCTAAC, I3sil (“silencer in the intron upstream of tandem 3′ splice sites”): CCAAGC, identified by Rosenberg et al. (2015) were introduced into the same positions as above, with the three splice site-proximal positions in the 9 nt windows left unchanged.

Secondary structure (subsets ending in “secvar” in Supplementary Data 5–8): For changing local secondary structures around splice sites, two insertion sites per splice site were defined (with a length of 9 nt, introduced in frame such that the 3 nt upstream and 15 nt downstream of donor splice sites and 28 nt upstream and 3 nt downstream of acceptor splice sites were not changed). There, either the complement or the reverse complement for sequences at least 3 nt away (to allow for hairpin formation) were introduced, specifically −24:−15, 0:+9 and +3: +12 for donor splice sites and −9:0, −12:−3, +16: +25/ + 17:+26 for acceptor splice sites, depending on the specific requirements imposed by the experimental design.

Recoding and CG/GC (subsets ending in “nuc” in Supplementary Data 5–8): Most native sequence contexts were recoded either by random choice of synonymous codons or selection of synonymous codons with the highest or lowest GC content. The following triplets in frame were left unchanged so to not interfere with the basic functionality of the splice site: Eleven triplets before and one triplet after an acceptor site, as well as two full triplets (at least 6 nt, depending on the coding frame) before and after a donor site. CG and GC dinucleotides were introduced at different frequencies, leaving the 28 nt before and at least 3 nt after an acceptor as well as at least 3 nt before and at least 7 nt after a donor site unchanged.

Combinatorial variants (subsets ending in “comb” and “combthreeway” in Supplementary Data 5–8): For 3–5 sets of contexts with equal intron or exon length or identical distance between two tandem splice sites (on average around six contexts in each set), all possible combinations of the three exonic, intronic or alternatively used exonic elements were created.

Introns and exons of identical length with no evidence for alternative splicing in RNAseq data (Encode, polyA RNA-seq of K562, Gingeras Lab, accession number ENCFF000HFA; Supplementary Data 9–12) were used to replace components of the alternative splice site contexts (subsets “comb_with_constitutive” in Supplementary Data 5–8). For retained introns, all possible combinations of upstream exon, intron and downstream exon in all 38 contexts were replaced with the corresponding sequences from on average 3 constitutively spliced introns and their surrounding exons. For five groups of cassette exons of identical length, with around six contexts in each group, all components and combinations thereof were replaced with 5–6 constitutive exons and their surrounding intronic regions. In the case of tandem 5′ and 3′ splice sites, eight exon-intron and eight intron-exon regions with no evidence for alternative donor or acceptor sites were used to replace the corresponding exonic and intronic parts in 51 and 48 sequence contexts, respectively.

### K562 cell culture

K562 cells were acquired from ATCC. Cells were grown in Iscove’s modified Dulbecco medium supplemented with 10% fetal bovine serum (SIGMA) and 1% Penicillin-Streptomycin solution (SIGMA). The cells were split when reaching a concentration of ~10^6^ cells/ml. The cells were grown in an incubator at 37 °C and 5% CO_2_. Cells were frozen in batches of 4 × 10^6^ cells in growth medium supplemented with 5% DMSO.

### Construction of the master plasmid

Master plasmids for library insertion were constructed by amplifying parts from the genomic DNA or already existing vectors and cloning the parts sequentially into pZDonor 3.1. The master plasmid for the retained intron library contained the EF1alpha promoter, mCherry, a designed multiple cloning site containing restriction sites for library cloning (RsrII and AscI) and for inserting a downstream fragment (XbaI), GFP and the SV40 terminator sequence. As the alternative 5′ splice sites library only contained donor sites, the 3′ end of the intron (149 nt) and the beginning of the downstream exon (100 nt) corresponding to the EIF2D context used in the library were amplified from K562 genomic DNA (using primers EIF2Dfor and EIF2Drev (Supplementary Data 13)) and cloned downstream of the library insertion site using AscI/XbaI. For the cloning of the cassette exon library, the 3′ end of the intron (722 nt) and the beginning of the exon (102 nt) downstream to a cassette exon in MCL1 were amplified from K562 genomic DNA (using primers MCL1downstreamfor and MCL1downstreamrev (Supplementary Data 13)) and cloned downstream of the library insertion site using AscI/XbaI. The full sequence of the transcribed library vectors including coordinates for mCherry and GFP coding regions and library insertion sites can be found in Supplementary Data 14.

### Synthetic library cloning

The cloning steps were performed essentially as described previously^27^. We used Agilent oligo library synthesis technology to produce a pool of 55,000 different fully designed single-stranded 210-oligomers (Agilent Technologies, Santa Clara, CA), which was provided as a single pool of oligonucleotides (10 pmol). The four subsets of this pool corresponding to the libraries tested here were defined by unique amplification primers (Supplementary Data 13). The pool of oligos was dissolved in 200 μl Tris-ethylenediaminetetraacetic acid (Tris-EDTA) and then diluted 1:50 with Tris-EDTA, which was used as template for PCR. We amplified each of the four libraries by performing eight PCR reactions, each of which contained 19 μl of water, 5 μl of DNA, 10 μl of 5 × Herculase II reaction buffer, 5 μl of 2.5 mM deoxynucleotide triphosphate (dNTPs) each, 5 μl of 10 μM forward primer, 5 μl of 10 μM reverse primer, and 1 μl Herculase II fusion DNA polymerase (Agilent Technologies). The parameters for PCR were 95 °C for 1 min, 14 cycles of 95 °C for 20 s, and 68 °C for 1 min, each, and finally one cycle of 68 °C for 4 min. The oligonucleotides were amplified using library-specific common primers in the length of 35 nt, which have 18-nt complementary sequence to the single-stranded 210-mers and a tail of 17 nt containing RsrII (forward primer) and AscI (reverse primer) restriction sites. The PCR products were concentrated using Amicon Ultra, 0.5 ml 30 K centrifugal filters (Merck Millipore). The concentrated DNA was then purified using a PCR mini-elute purification kit (Qiagen) according to the manufacturer’s protocol. Purified library DNA (540 ng total) was cut with the unique restriction enzymes RsrII and AscI (Fermentas FastDigest) for 2 h at 37 °C in two 40-μl reactions containing 4 μl fast digest (FD) buffer, 1 μl RsrII enzyme, 1 μl AscI enzyme, 18 μl DNA (15 ng/μl), and 16 μl water, followed by heat inactivation for 20 min at 65 °C. Digested DNA was separated from smaller fragments and uncut PCR products by electrophoresis on a 2.5% agarose gel stained with GelStar (Cambrex Bio Science Rockland). Fragments were cut from the gel and eluted using electroelution Midi GeBAflex tubes (GeBA, Kfar Hanagid, Israel). Eluted DNA was precipitated using sodium acetate–isopropanol. The master plasmids were cut with RsrII and AscI (Fermentas FastDigest) in a reaction mixture containing 6 μl FD buffer, 3 μl of each enzyme and 3.5 μg of the plasmid in a total volume of 60 μl. After incubation for 2.5 h at 37 °C, 3 μl FD buffer, 3 μl alkaline phosphatase (Fermentas) and 24 μl water were added and the reactions were incubated for an additional 30 min at 37 °C followed by 20 min at 65 °C. Digested DNA was purified using a PCR purification kit (Qiagen). The digested plasmids and DNA library were ligated for 30 min at room temperature in a 10 μl reactions, containing 150 ng plasmid and the library in a molar ratio of 1:1, 1 μl CloneDirect 10 × ligation buffer, and 1 μl CloneSmart DNA ligase (Lucigen Corporation), followed by heat inactivation for 15 min at 70 °C. Ligated DNA was transformed into *E. coli* 10 G electrocompetent cells (Lucigen) divided into aliquots (23 µl each, plus 2 μl of the ligation mix), which were then plated on 4 Luria broth (LB) agar (200 mg/ml amp) 15-cm plates per transformation reaction (25 μl). For each library between 2 and 4 transformation reactions were performed. We collected between 0.5 × 10^6^ and 1.5 × 10^6^ colonies per library the day after transformation by scraping the plates into LB medium. Library-pooled plasmids were purified using a NucleoBond Xtra maxi kit (Macherey Nagel). To ensure that the collected plasmids contain only a single insert of the right size, we performed colony PCR (at least 16 random colonies per single transformation reaction).

For alternative 3′ splice sites and cassette exons, a common upstream donor site had to be introduced. To enable unambiguous identification of the variants based on the barcode at the 5′ end of the variable region, this had to be carried out after cloning of the library, as otherwise the 5′ end of the inserted library variants would be located in an intron and undetectable on the level of spliced mRNAs. In the case of alternative 3′ splice sites, the upstream exon (38 nt) and the 5′ end of the intron (132 nt) corresponding to the STAT3 context used in the library were amplified from K562 genomic DNA (using primers STAT3for and STAT3rev (Supplementary Data 13)) and cloned into the library using AscI/XbaI, following the same protocol as above for the cloning of the oligonucleotide libraries. For the cloning of the cassette exon library, the upstream exon (52 nt) and the 5′ end of the intron (224 nt) upstream of the cassette exon in MCL1 from which the downstream sequences had been taken were amplified from K562 genomic DNA (using primers MCL1upstreamfor and MCL1upstreamrev (Supplementary Data 13)) and cloned into the library using AscI/XbaI (see Supplementary Fig. 1A and Supplementary Data 14).

### Transfection into K562 cells and genomic integration

The purified plasmid library was transfected into K562 cells and genomically integrated using the Zinc Finger Nuclease (ZFN) system for site-specific integration and the CompoZr® Targeted Integration Kit - AAVS1 (SIGMA). Transfections were carried out using Amaxa® Cell Line Nucleofector® Kit V (LONZA). To ensure library representation we performed 10 nucleofections of the purified plasmid library. For each nucleofection, 4 × 10^6^ cells were centrifuged and washed twice with 20 ml of Hank’s balanced salt solution (HBSS, SIGMA). Cells were resuspended in 100 μl solution (warmed to room temperature) composed of 82 μl solution V and 19 μl supplement (Amaxa® Cell Line Nucleofector® Kit V). Next, the cells were mixed with 2.75 μg of donor plasmid and 0.6 μg ZFN mRNA (prepared in-house) just prior to transfection. Nucleofection was carried out using program T-16 on the Nucleofector^TM^ device, immediately mixed with ~0.5 ml of pre-cultured growth medium and transferred to a 6-well plate with additional 1.5 ml of pre-cultured growth medium. A purified plasmid library was also transfected without the addition of ZFN and served as a control to determine when cells lost non-integrated plasmids.

### Sorting the library by FACS

K562 cells were grown for at least 14 days to ensure that non-integrated plasmid DNA was eliminated. A day prior to sorting, cells were split to ~0.25 × 10^6^ cells/ml. On the day of sorting, cells were centrifuged, resuspended in sterile PBS and filtered using cell-strainer capped tubes (Becton Dickinson (BD) Falcon). Sorting was performed with BD FACSAria II SORP (special-order research product) at low sample flow rate and a sorting speed of ~18,000 cells/s. To sort cells that integrated the reporter construct successfully and in a single copy (~4% of the population), we determined a gate according to mCherry fluorescence so that only mCherry-expressing cells corresponding to a single copy of the construct were sorted (mCherry single population). We collected a total of 3.1–3.9 × 10^6^ cells for each library (around 350 cells/variant on average) in order to ensure adequate library representation.

In the case of the retained introns library, cells sorted for single integration of the transgene were grown for a week before we sorted the population into 16 bins according to the GFP/mCherry ratio. Each bin was defined to span a range of GFP/mCherry ratio values such that it contains between 1 and 10% of the cell population. We collected a total of 1.2 × 10^7^ cells in order to ensure adequate library representation (>1000 cells/variant on average). Cells from each bin were grown separately for freezing and purification of genomic DNA.

### RNA purification, cDNA synthesis, and sample preparation

For the cell population sorted for single integration of the reporter construct we performed RNA purification by centrifuging 10^7^ cells, washing them with PBS, splitting into two tubes and purifying RNA using NucleoSpin RNA II kit (Macherey-Nagel) according to the manufacturer’s protocol. We prepared cDNA in four reverse transcription reaction for each replicate using SuperScript® III First-Strand Synthesis System (Thermo Fisher Scientific) with random hexamer primers and 5 μg of input RNA (per reaction) according to the manufacturer protocol. For amplification of the library variants, three PCR reactions of 50 μl total volume were performed. Each reaction contained 5 μl cDNA, 25 μl of Kapa Hifi ready mix X2 (KAPA Biosystems), 2.5 μl 10 μM 5′ primer, and 2.5 μl 10 μM 3′ primer. The PCR program was 95 °C for 5 min, 20 cycles of 94 °C for 30 s and 72 °C for 30 s, each, and one cycle of 72 °C for 5 min. Specific primers corresponding to the constant region upstream and downstream of the splice sites were used (Supplementary Data 13). The PCR products were separated from potential unspecific fragments by electrophoresis on a 1.5% agarose gel stained with EtBr, cut from the gel, and cleaned in two steps: gel extraction kit (Qiagen) and SPRI beads (Agencourt AMPure XP). The sample was assessed for size and purity at the Tapestation, using high sensitivity D1K screenTape (Agilent Technologies, Santa Clara, California). We used 20 ng library DNA for library preparation for NGS; specific Illumina adaptors were added, and DNA was amplified using 14 amplification cycles. The sample was reanalyzed using Tapestation.

### Genomic DNA isolation, amplification, and sample preparation

For each of the 16 bins of the retained intron library we purified genomic DNA by centrifuging 5 × 10^6^ cells, washing them with 1 ml PBS and purifying DNA using DNeasy Blood & Tissue Kit (Qiagen) according to the manufacturer’s protocol. In order to maintain the complexity of the library amplified from gDNA, PCR reactions were carried out on a gDNA amount calculated to contain a minimum average of 200 copies of each oligo included in the sample. For each of the 16 bins, we used 15 µg of gDNA as template in a two-step nested PCR. In the first step, three reactions were performed and each reaction contained 5 μg gDNA, 25 μl Kapa Hifi ready mix X2 (KAPA Biosystems), 2.5 μl 10 μM 5′ primer, and 2.5 μl of 10 μM 3′ primer (Supplementary Data 13). The parameters for the first PCR were 95 °C for 5 min, 18 cycles of 94 °C for 30 s, 65 °C for 30 s, and 72 °C for 60 s, each, and one cycle of 72 °C for 5 min. In the second PCR step, each reaction contained 2.5 μl of the first PCR product, 25 μl of Kapa Hifi ready mix X2 (KAPA Biosystems), 2.5 μl 10 μM 5′ primer, and 2.5 μl 10 μM 3′ primer. The PCR program was 95 °C for 5 min, 24 cycles of 94 °C for 30 s and 72 °C for 30 s, each, and one cycle of 72 °C for 5 min. Specific primers corresponding to the constant region of the plasmid were used (Supplementary Data 13). The 5′ primer contained a unique upstream 8-nt bin barcode sequence, and three different barcodes were used for each bin. The 3′ primer was common to all bins. Multiple PCR reaction products of each bin were combined. The concentration of the PCR samples was measured using a monochromator (Tecan i-control), and the samples were mixed in ratios corresponding to their ratio in the population, as defined when sorting the cells into the 16 bins. Sample preparation including gel elution and purification were performed as described above for amplicons from cDNA.

### Mapping next generation sequencing reads

To unambiguously identify the variant of origin, a unique 12-mer barcode sequence was placed at the 5′ end of each variable region. DNA was sequenced on a NextSeq-500 sequencer. For cDNA we obtained 14.5, 4.3, 13.3, and 40.1 million reads for the retained intron, cassette exon, tandem 5′ and tandem 3′ libraries, respectively (2 × 150PE). Reads were first assigned according to their barcode (read 1) and subsequently the exact position of splicing (or lack thereof) was mapped using the corresponding mate (read 2) and assigned to either of the splice variants or, in the case of even a single mismatch or usage of a cryptic splice site, discarded. Both steps were performed using custom-made Python scripts.

For amplicons from genomic DNA from the 16 bins, into which the retained intron library was sorted, we obtained a total of ~12 million paired end reads (2 × 150 bp), in order to cover the entire length of the variable region, not only the barcode, to filter out mutations introduced during synthesis or cloning, which could distort the protein readout (especially in the case of nonsense mutations and indels). Using Python scripts (see Code availability statement) we determined for each read its bin barcode and its variant barcode and discarded all the reads that could not be assigned to a bin and a library variant of origin or contained even a single mismatch anywhere along the full length of the variant.

### Computing RNA splicing ratios

For all variants with at least 100 reads mapped we computed the log2 ratio of spliced/unspliced reads for retained introns, exon included/exon skipped for cassette exons and downstream splice site used/upstream splice site used for tandem 5′ and 3′ splice site libraries, and refer to this throughout the text and Figures as “splicing ratio” (in log2). A splicing ratio of 0 therefore indicates an equal number of reads mapping to the two possible splicing outcomes, with a positive value indicating more reads mapping to the spliced/“exon included”/“downstream splice site used” isoform and a negative value indicating more reads mapping unspliced/“exon skipped”/“upstream splice site used” isoform. In cases where more than 100 reads were mapped to a given variant, but all of them represented the same isoform, we added one read to the count of either isoform in order to enable us to calculate the log ratio for these variants. We chose to present splicing ratio as the ratio between the two expected outcomes, as opposed to PSI (percent spliced-in) in order to have a measure that is meaningful across all splicing types tested here. In addition the log ratio between the two splice variants results in a larger dynamic range close to extreme values (0 or 100% spliced-in, i.e., dominance of one isoform). The small variance within barcode control groups around these values shows that our assay indeed is quantitative enough to draw conclusions even in this range.

After filtering we obtained RNA splicing ratios for 6626 (77.5%), 7249 (75.4%), 5266 (70.5%), and 4828 (67.5%) of the variants for the four libraries, respectively.

To determine normalized splicing ratios (i.e., the paired difference of a variant to the corresponding wild-type context) we first calculated a mean splicing value (or noise value) for each context from triplicates (with different barcodes) added for all of the wild-type contexts. We then subtracted the corresponding mean wild-type level from each of the variants’ splicing values.

### Computing protein splicing values

We applied a number of filters to the raw sequencing data to reduce experimental noise. First, variants with <200 reads mapped across bins were removed. Second, for bins with a read count of less than five or bins that got <2% of overall reads, the bin value was set to zero. Third, for each variant we set to zero bins surrounded by zero values (isolated bins). Forth, for each variant we set all cells to zero if the sum of normalized reads after filtering was <30% of the sum of normalized reads before filtering. For each variant, we normalized the values across the 16 bins and applied a Savitzky-Golay filter for smoothing the data. We detected peaks in the smoothed vector by a simple approach in which a point is considered a maximum peak if it has the maximal value, and was preceded (to the left) by a value lower by delta (which we set to 0.05). Variants with no or more than one peak after smoothing were disregarded in all protein-based analyses.

For each bin, we calculated the median of the log2 of GFP/mCherry as measured by FACS for all the cells sorted into that bin. For each variant, we calculated the weighted average and the variance for the distribution of reads across bins (using unsmoothed read counts normalized for each variant and taking the median of GFP/mCherry ratios of cells sorted into one bin as the value associated with this bin), resulting in what is referred to in the main text and Figures as the “splicing value” (in log2) and, by dividing the variance by the mean, the “noise strength”. After filtering we obtained protein-based splicing values for 73% of the variants from our library of retained introns and 56% of the variants from our library of tandem 5′ splice sites. Noise residuals were calculated by fitting a generalized additive model to the relationship between noise strength and splicing value (spline term) and RNA expression (RNA/DNA reads; linear term) using the pygam package (version 0.8.0) and calculating the deviation of each point from this line.

### Machine learning approaches

All machine learning procedures were carried out using the python sklearn package (version 0.18.2). Initially, from all duplicated sequences (e.g., barcode control sets), which passed filtering, a single variant was randomly chosen for all subsequent steps to avoid biases resulting from having duplicated sequences. Ten percent of the remaining variants were put aside and used only for evaluation of models built using the other 90%. We chose Gradient Boosting Regression as the prediction algorithm because it can capture non-linear interactions between features, which is especially relevant in the case of a complex problem like splicing prediction with many positional and combinatorial effects known.

For prediction based on hexamers, we counted the number of occurrences of every possible hexamer separately in the upstream exon, intron and downstream exon for retained introns, the upstream intron, exon and downstream intron for cassette exons and the exon, alternative exon and intron for tandem 5′ and 3′ splice sites, restricting ourselves to the designed variable region and disregarding the barcode (except for the case of the retained intron library where the barcode region was included as it was relatively close (minimal distance 20 nt) from the donor splice site in a number of variants).

For prediction based on RBP binding sites, we used position weight matrices of RBP binding sites from the ATtRACT database^19^ to calculate the sum of log-odds ratios for all potential binding sites separately in the upstream exon, intron and downstream exon for retained introns, the upstream intron, exon and downstream intron for cassette exons and the exon, alternative exon and intron for tandem 5′ and 3′ splice sites, restricting ourselves to the designed variable region and disregarding the barcode (except for the case of the retained intron library where the barcode region was included as it was relatively close (minimal distance 20 nt) from the donor splice site in a number of variants). Initially, we selected for all human motifs in the database and subsequently let the model choose the most informative set of features (see description of feature selection below).

For secondary structure predictions we used the fold function from the Vienna RNA package 2.0 and extracted both the minimal free energy and the predicted pairedness for each position.

Different hyperparameter settings for learning rate, n_estimators, and max_depth were tested in a systematic and combinatorial fashion using 10-fold cross-validation. Typically around 100 tests were performed and the best set of hyperparameters used for subsequent steps.

Feature selection was performed using optimized hyperparameters and sklearn’s feature_selection.SelectFromModel function. Another hyperparameter optimization step was performed to ensure that the previously chosen hyperparameters were still optimal for the reduced set of features.

At the end, the model was evaluated by training it on the entire training set (90% of all relevant unique library variants) and scoring the accuracy of prediction based on the held-out test set (10% of relevant unique library variants), which had not been used at any stage during development of the model. The *R*^2^ (coefficient of determination, calculated using the sklearn function metrics.r2_score) regression score and the Pearson correlation coefficient (as calculated using scipy.stats.pearsonr) were chosen as a measure.

Feature importance and effect on the model was determined using SHAP analysis^35,36^.

### Testing the model on data from other reporter assays

To test the performance of our model on other datasets we extracted the features relevant for our predictive model from the DNA sequences (variable region and context) used in three other studies^8,10,24^. All data from these studies were obtained through github.com/gagneurlab/MMSplice_paper to make our input comparable to the one used by Cheng et al.^25^. To compute the effect of sequence variants on splicing ratio we calculated the predicted (log) splicing ratios based on wild-type and mutant sequence separately and report the difference between those pairs. We compare the performance of our model to a recent study^25^ testing state-of-the-art predictors on the same datasets (Vex-seq and MaPSy). In all tests on other datasets we report the Pearson and Spearman correlation coefficients and not the *R*^2^ (coefficient of determination, calculated using the sklearn function metrics.r2_score) regression score because the assays are performed in different ways, systematically affecting measured splicing ratios and making the comparison of absolute values and thereby the *R*^2^ regression score less informative.

### General data analysis

For data analysis, we used python 2.7.11 with pandas 0.20.3, numpy 1.13.1, seaborn 0.6, scipy 0.17, pygam 0.8.0, sklearn 0.18.2, and shap 0.28.5. Confidence intervals were calculated by bootstrapping (1000 iterations).

### Reporting summary

Further information on research design is available in the Nature Research Reporting Summary linked to this article.

## Supplementary information

Supplementary Information

Reporting Summary

Peer Review

Description of Additional Supplementary Files

Supplementary Data 1

Supplementary Data 2

Supplementary Data 3

Supplementary Data 4

Supplementary Data 5

Supplementary Data 6

Supplementary Data 7

Supplementary Data 8

Supplementary Data 9

Supplementary Data 10

Supplementary Data 11

Supplementary Data 12

Supplementary Data 13

Supplementary Data 14

## Data Availability

A reporting summary for this Article is available as a Supplementary Information file. All sequencing data generated in this study are available in the NCBI gene expression omnibus (GEO) under accession GSE132064. All data are available from the corresponding author upon reasonable request.

## Code availability

The code used to process the data, generate the Figures and train and test the predictive models are available as a GitHub repository (https://github.com/martinmikl/splicing_mpra).
