# Supplementary material for: Dissecting splicing decisions and cell-to-cell variability with designed sequence libraries
Source: Nat Commun. 2019 Oct 8;10:4572. doi: 10.1038/s41467-019-12642-3 (PMC6783452; doi:10.1038/s41467-019-12642-3)
Supplement: Supplementary file 1 — Supplementary Information [file 41467_2019_12642_MOESM1_ESM.pdf]

Supplementary Information

Dissecting splicing decisions and cell-to-cell variability with designed sequence libraries

Mikl et al.

Correspondence to: eran.segal@weizmann.ac.il, martin.mikl@weizmann.ac.il

This PDF file includes:

Supplementary Figures 1-8

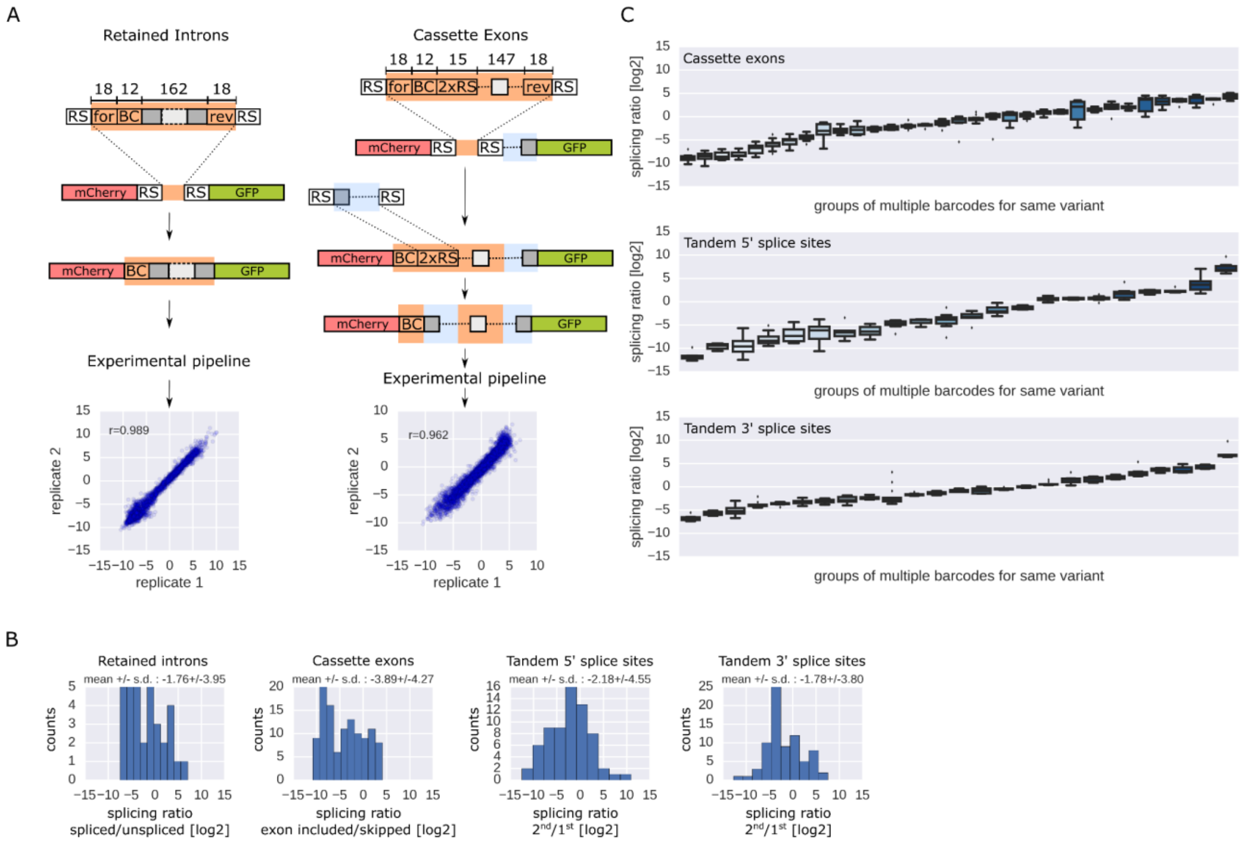

Supplementary Figure 1. Cloning strategy for the library and reproducibility of the assay.

A. Structure and cloning strategy for retained introns and cassette exons (orange=part of the designed and synthesized oligonucleotide, light blue=constant region, in this case containing upstream exon, donor site and intron and downstream intron, acceptor site and exon, respectively; numbers indicate length in base pairs; RS=restriction site, for=forward primer, rev=reverse primer, BC=barcode), with correlation between technical replicates (cells were taken from the same pool, all the subsequent steps starting from collection of the cells were performed independently). B. Distribution of splicing ratios (as determined in our assay) of the sets of native splice site sequences used as library contexts (s.d.: standard deviation). C. Barcode controls for cassette exon and tandem 5' and 3' splicing ratios, boxplots for groups of multiple barcodes for the same sequence variant (n>7 for all groups), plotted according to their mean splicing ratio; boxes show the quartiles of the dataset, whiskers show the range of the distribution not including outliers (displayed as points).

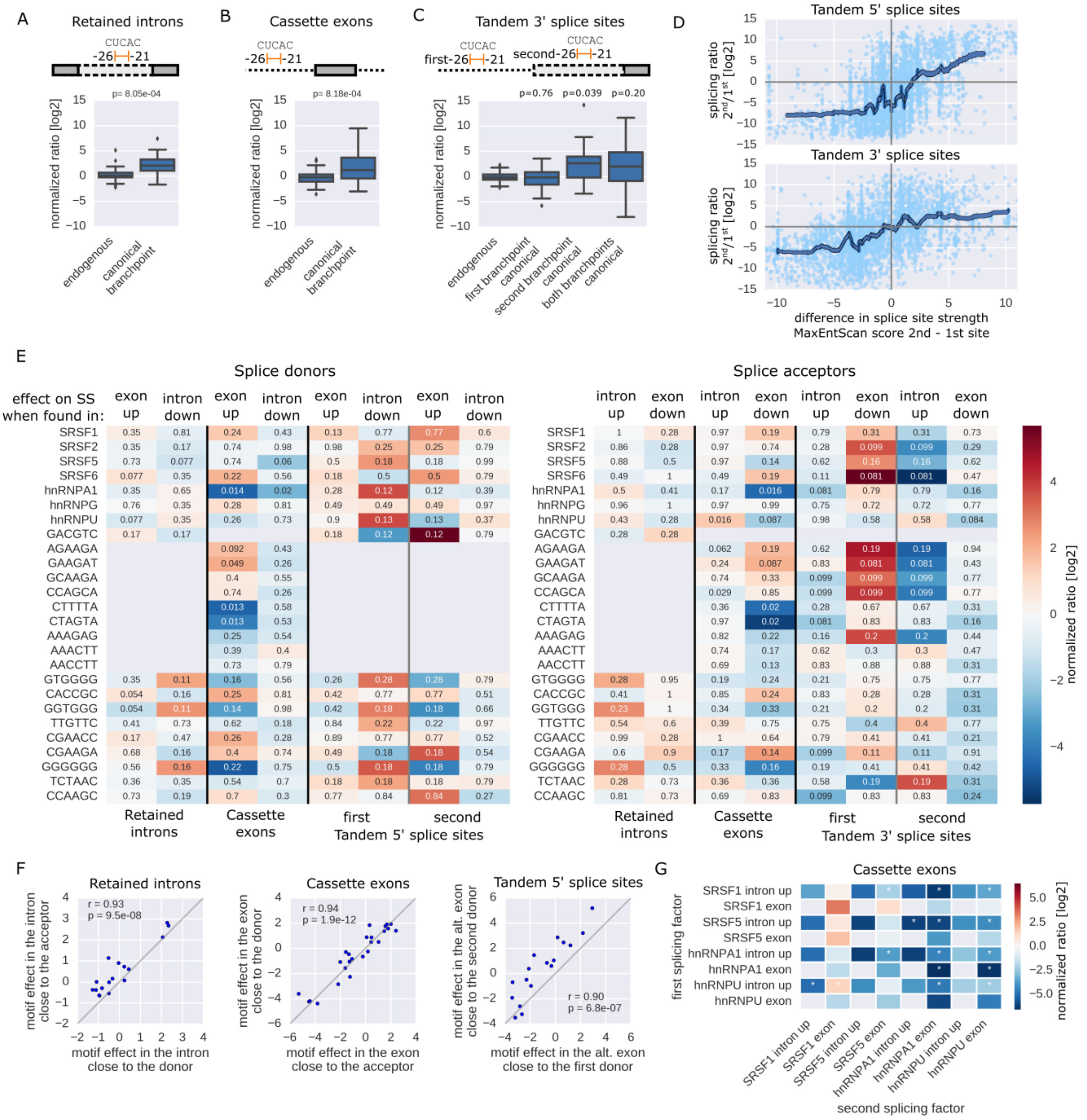

A
Retained introns
CUCAC
-26 -21
p= 8.05e-04
normalized ratio [log2]
endogenous
canonical branchpoint
B
Cassette exons
CUCAC
-26 -21
p= 8.18e-04
normalized ratio [log2]
endogenous
canonical branchpoint
C
Tandem 3' splice sites
CUCAC
first-26 -21
CUCAC
second-26 -21
p=0.76 p=0.039 p=0.20
normalized ratio [log2]
endogenous
first branchpoint canonical
second branchpoint canonical
both branchpoints canonical
D
Tandem 5' splice sites
Tandem 3' splice sites
splicing ratio 2nd/1st [log2]
difference in splice site strength
MaxEntScan score 2nd - 1st site
E
Splice donors
Splice acceptors
effect on SS when found in:
exon up
intron down
intron up
exon down
SRSF1
SRSF2
SRSF5
SRSF6
hnRNPA1
hnRNPG
hnRNPU
GACGTC
AGAAGA
GAAGAT
GCAAGA
CCAGCA
CTTTTA
CTAGTA
AAAGAG
AAACTT
AACCTT
GTGGGG
CACCGC
GGTGGG
TTGTTC
CGAACC
CGAAGA
GGGGGG
TCTAAC
CCAAGC
Retained introns
Cassette exons
first second Tandem 5' splice sites
first second Tandem 3' splice sites
normalized ratio [log2]
F
Retained introns
r = 0.93
p = 9.5e-08
motif effect in the intron close to the acceptor
motif effect in the intron close to the donor
Cassette exons
r = 0.94
p = 1.9e-12
motif effect in the exon close to the donor
motif effect in the exon close to the acceptor
Tandem 5' splice sites
r = 0.90
p = 6.8e-07
motif effect in the alt. exon close to the second donor
motif effect in the alt. exon close to the first donor
G
Cassette exons
SRSF1 intron up
SRSF1 exon
SRSF5 intron up
SRSF5 exon
hnRNPA1 intron up
hnRNPA1 exon
hnRNPU intron up
hnRNPU exon
first splicing factor
second splicing factor
normalized ratio [log2]

Supplementary Figure 2. Effects of splice site mutations and splicing factor binding sites on splicing.

A-C. Normalized ratios for variants with endogenous intronic sequences vs. variants in which a canonical branchpoint sequence (CTCAC) has been introduced at position –25 relative to the acceptor splice site (n=14-45 for individual groups); p-values were determined using Wilcoxon signed-rank test; boxes show the quartiles of the dataset, whiskers show the range of the distribution not including outliers (displayed as points). D. Splicing ratio for all tandem 5' or 3’ splice site variants, plotted against the difference in splice site strength (second – first) as calculated using the MaxEntScan package. The dark blue line denotes the running average (window size 300) with confidence intervals (as determined by bootstrapping). E. Heatmap of mean effects on usage of the adjacent splice site of introducing sequence motifs in the indicated libraries and locations. 5’ and 3’ insertion positions in retained introns, cassette exons and the alternative exon between 5’ tandem splice sites were treated independently and assigned either as influencing the adjacent acceptor or donor splice site. In the case of tandem 3’ splice site insertion locations were not split according to their proximity to either the first or second splice sites due to the low number of possible insertion locations (1-2), but both were assumed to potentially influence the upstream and downstream acceptor. p-values were determined using Wilcoxon signed-rank test and corrected for multiple testing (Benjamini-Hochberg) for each splicing type separately. F. Effect of introducing a motif either towards the 5’ or 3’ end of retained introns, cassette exons and the alternative exon between tandem 5’ splice sites. G. Heatmap of mean effects of introducing a combination of two splicing factor binding sites at the indicated locations. Asterisks denote cases where the trend reached statistical significance ($p<0.05$, Wilcoxon signed-rank test, n=3-22 for each combination).

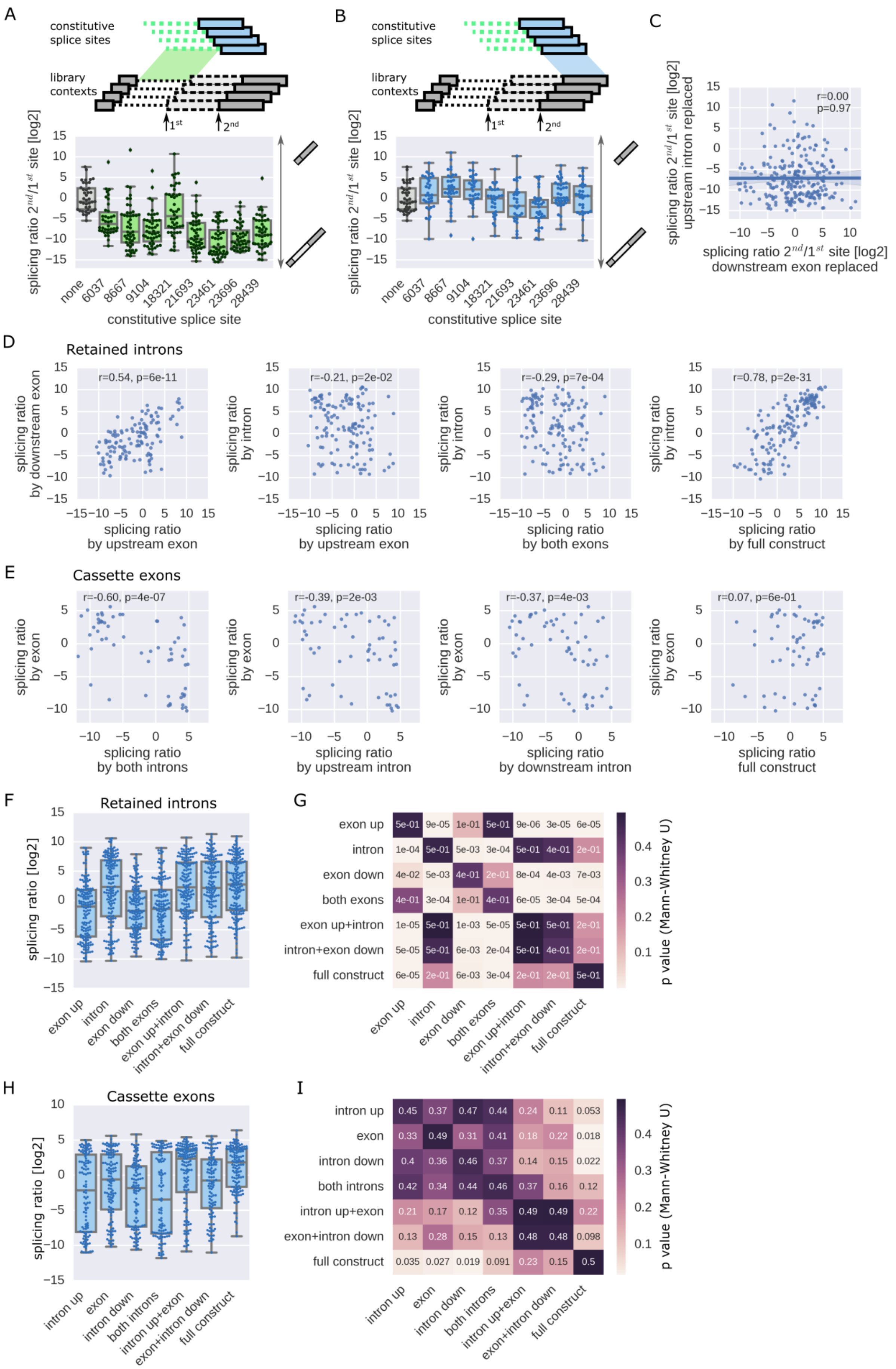
A
constitutive splice sites
library contexts
1st
2nd
splicing ratio 2nd/1st site [log2]
none
6037
8667
9104
18321
21693
23461
23696
28439
constitutive splice site
B
constitutive splice sites
library contexts
1st
2nd
splicing ratio 2nd/1st site [log2]
constitutive splice site
C
r=0.00
p=0.97
splicing ratio 2nd/1st site [log2] upstream intron replaced
splicing ratio 2nd/1st site [log2] downstream exon replaced
D
Retained introns
r=0.54, p=6e-11
splicing ratio by downstream exon
splicing ratio by upstream exon
r=-0.21, p=2e-02
splicing ratio by intron
splicing ratio by upstream exon
r=-0.29, p=7e-04
splicing ratio by intron
splicing ratio by both exons
r=0.78, p=2e-31
splicing ratio by intron
splicing ratio by full construct
E
Cassette exons
r=-0.60, p=4e-07
splicing ratio by exon
splicing ratio by both introns
r=-0.39, p=2e-03
splicing ratio by exon
splicing ratio by upstream intron
r=-0.37, p=4e-03
splicing ratio by exon
splicing ratio by downstream intron
r=0.07, p=6e-01
splicing ratio by exon
splicing ratio full construct
F
Retained introns
splicing ratio [log2]
exon up
intron
exon down
both exons
exon up+intron
intron+exon down
full construct
G
exon up: 5e-01 9e-05 1e-01 5e-01 9e-06 3e-05 6e-05
intron: 1e-04 5e-01 5e-03 3e-04 5e-01 4e-01 2e-01
exon down: 4e-02 5e-03 4e-01 2e-01 8e-04 4e-03 7e-03
both exons: 4e-01 3e-04 1e-01 4e-01 6e-05 3e-04 5e-04
exon up+intron: 1e-05 5e-01 1e-03 5e-05 5e-01 5e-01 2e-01
intron+exon down: 5e-05 5e-01 6e-03 2e-04 5e-01 4e-01 2e-01
full construct: 6e-05 2e-01 6e-03 3e-04 2e-01 2e-01 5e-01
p value (Mann-Whitney U)
H
Cassette exons
splicing ratio [log2]
intron up
exon
intron down
both introns
intron up+exon
exon+intron down
full construct
I
intron up: 0.45 0.37 0.47 0.44 0.24 0.11 0.053
exon: 0.33 0.49 0.31 0.41 0.18 0.22 0.018
intron down: 0.4 0.36 0.46 0.37 0.14 0.15 0.022
both introns: 0.42 0.34 0.44 0.46 0.37 0.16 0.12
intron up+exon: 0.21 0.17 0.12 0.35 0.49 0.49 0.22
exon+intron down: 0.13 0.28 0.15 0.13 0.48 0.48 0.098
full construct: 0.035 0.027 0.019 0.091 0.23 0.15 0.5
p value (Mann-Whitney U)

Supplementary Figure 3. Coordination and antagonism between genetic building blocks.

AB. Distribution of splicing ratios (2nd/1st) in tandem 3' splice sites in variants with the upstream intron (A, green) or the downstream exon (B, blue) replaced with the corresponding exonic or intronic part from a set of eight constitutive 3’ splice sites; gray box plots and data points denote wild type splicing ratios; boxes show the quartiles of the dataset, whiskers show the range of the distribution not including outliers (displayed as points). C. Each data point represents the log-ratio (2nd/1st splice site) for a pair of exonic (x-axis) and intronic (y-axes) from the same native constitutive donor site placed in a library context (based on the same data as the box plots in Fig S3AB). D-E. Each datapoint represents the splicing ratio for a context (containing a retained intron (D) or a cassette exon (E)), in which one of the indicated components has been replaced by the corresponding element from the same (unrelated) native splice site. Full construct indicates that all three components have been replaced with sequences from the same native splice site. Pearson correlation coefficient with the associated p-values are stated for each comparison. F. Distribution of splicing ratios of retained introns in which the indicated component is replaced with a component from a set of constitutive splice sites; boxes show the quartiles of the dataset, whiskers show the range of the distribution not including outliers (displayed as points). G. p-values (as determined using the Mann-Whitney U test) for the difference between all pairwise combinations of groups in F. H. Distribution of splicing ratios of cassette exons in which the indicated component is replaced with a component from a set of constitutive splice sites; boxes show the quartiles of the dataset, whiskers show the range of the distribution not including outliers (displayed as points). I. p-values (as determined using the Mann-Whitney U test) for the difference between all pairwise combinations of groups in H.

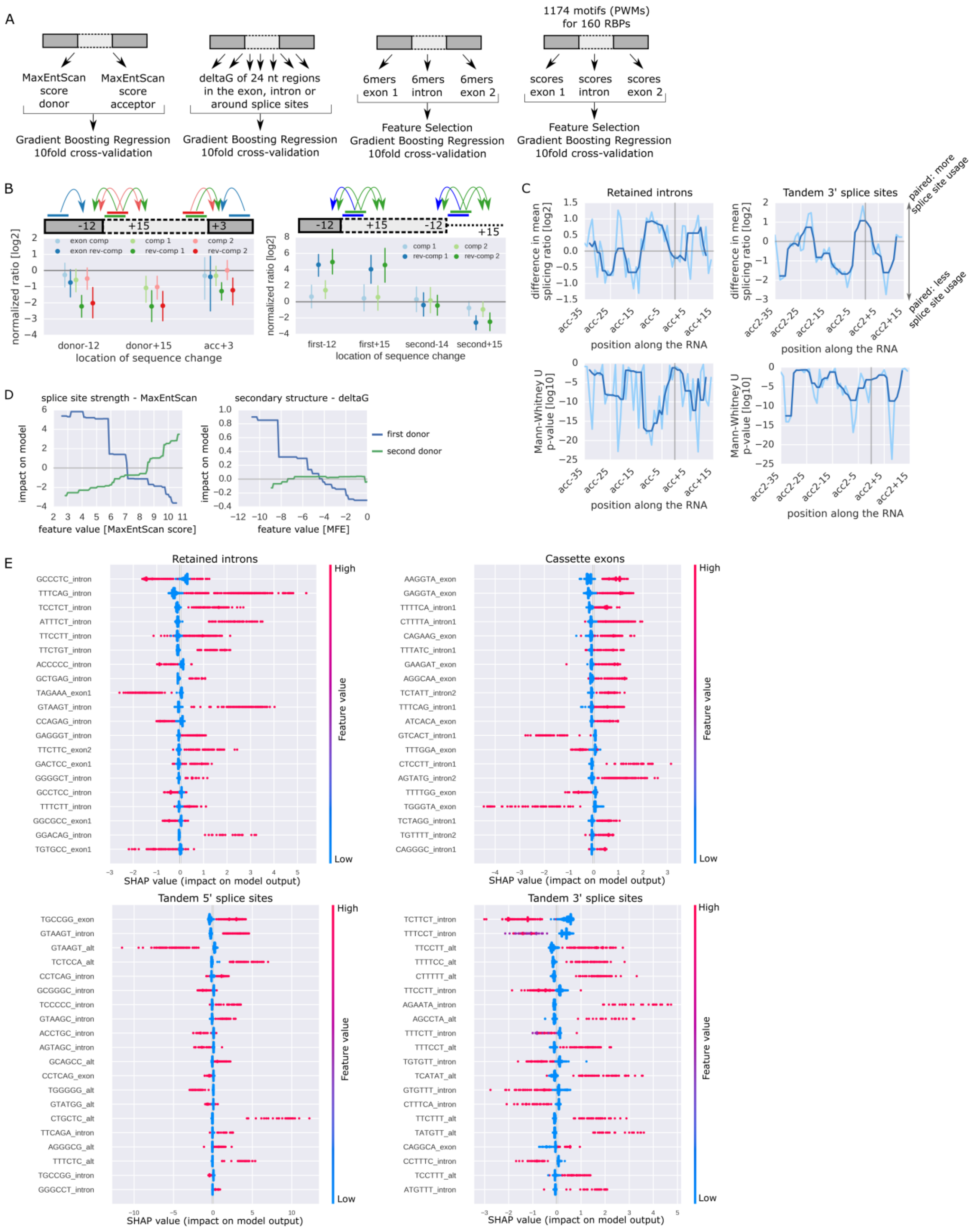
A
1174 motifs (PWMs) for 160 RBPs
MaxEntScan score donor
MaxEntScan score acceptor
deltaG of 24 nt regions in the exon, intron or around splice sites
6mers exon 1
6mers intron
6mers exon 2
scores exon 1
scores intron
scores exon 2
Feature Selection
Gradient Boosting Regression
10fold cross-validation
B
normalized ratio [log2]
location of sequence change
C
Retained introns
Tandem 3' splice sites
difference in mean splicing ratio [log2]
Mann-Whitney U p-value [log10]
position along the RNA
paired: more splice site usage
paired: less splice site usage
D
splice site strength - MaxEntScan
secondary structure - deltaG
impact on model
feature value [MaxEntScan score]
feature value [MFE]
first donor
second donor
E
Retained introns
Cassette exons
Tandem 5' splice sites
Tandem 3' splice sites
SHAP value (impact on model output)
Feature value
High
Low

Supplementary Figure 4. Feature generation for developing a predictive model for splicing.

A. Outlines for building a regressor model based on MaxEntScan scores for both splice sites, on minimum free energy (delta G) of 24 nt regions across the variable region, on hexamer counts in different parts of the variable region and on 1174 PWM scores for 160 RBPs in different parts of the variable region (shown for retained introns). B. Schematic depicting secondary structure manipulations in the retained intron (left) and tandem 5' splice sites (right) library. Blue, green and red lines indicate sequences whose complement or reverse complement is introduced in the indicated positions, corresponding to the color scheme in the plot below, where mean and 95% CI for the effect on the wild-type splicing ratio of introducing different sequence elements are shown for the indicated locations (n=19-28 for each group). C. Mean difference in splicing ratio between variants in which the indicated position along the x-axis is predicted to be paired and variants in which it is predicted to be unpaired (top) and the associated p-values (Mann-Whitney U) for the comparison between the two groups (position paired vs. not paired) for each indicated position relative to the splice sites (bottom). The darker blue line represents the rolling average of the surrounding five positions. D. The impact of splice site strength (left) and secondary structure around the donor sites (right) on the model prediction (as determined using SHAP) is plotted against the feature values. E. Hexamer feature effects (as determined using SHAP) on the model prediction (ranked by their importance for the prediction) for the different splicing type libraries. The color denotes the feature value (hexamer counts) and the position along the x-axis denotes the impact on model output, for each item in the training set.

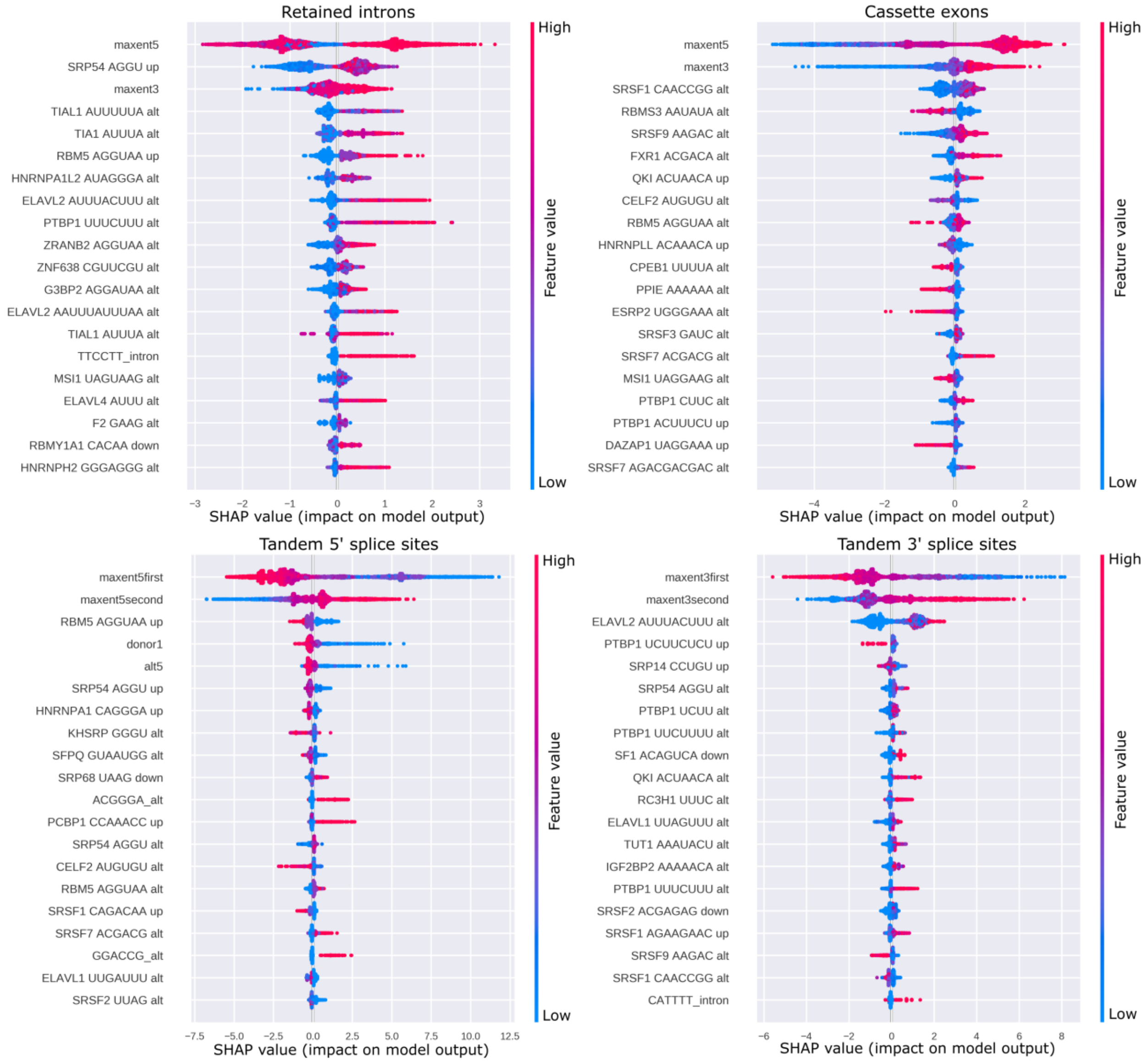

Supplementary Figure 5. Feature effects on model output.

A. Feature effects (as determined using SHAP) on the model prediction (ranked by their importance for the prediction) for the different splicing type libraries. The color denotes the feature value (MaxEntScan scores, deltaG, hexamer counts and cumulative binding scores) and the position along the x-axis denotes the impact on model output, for each item in the training set.

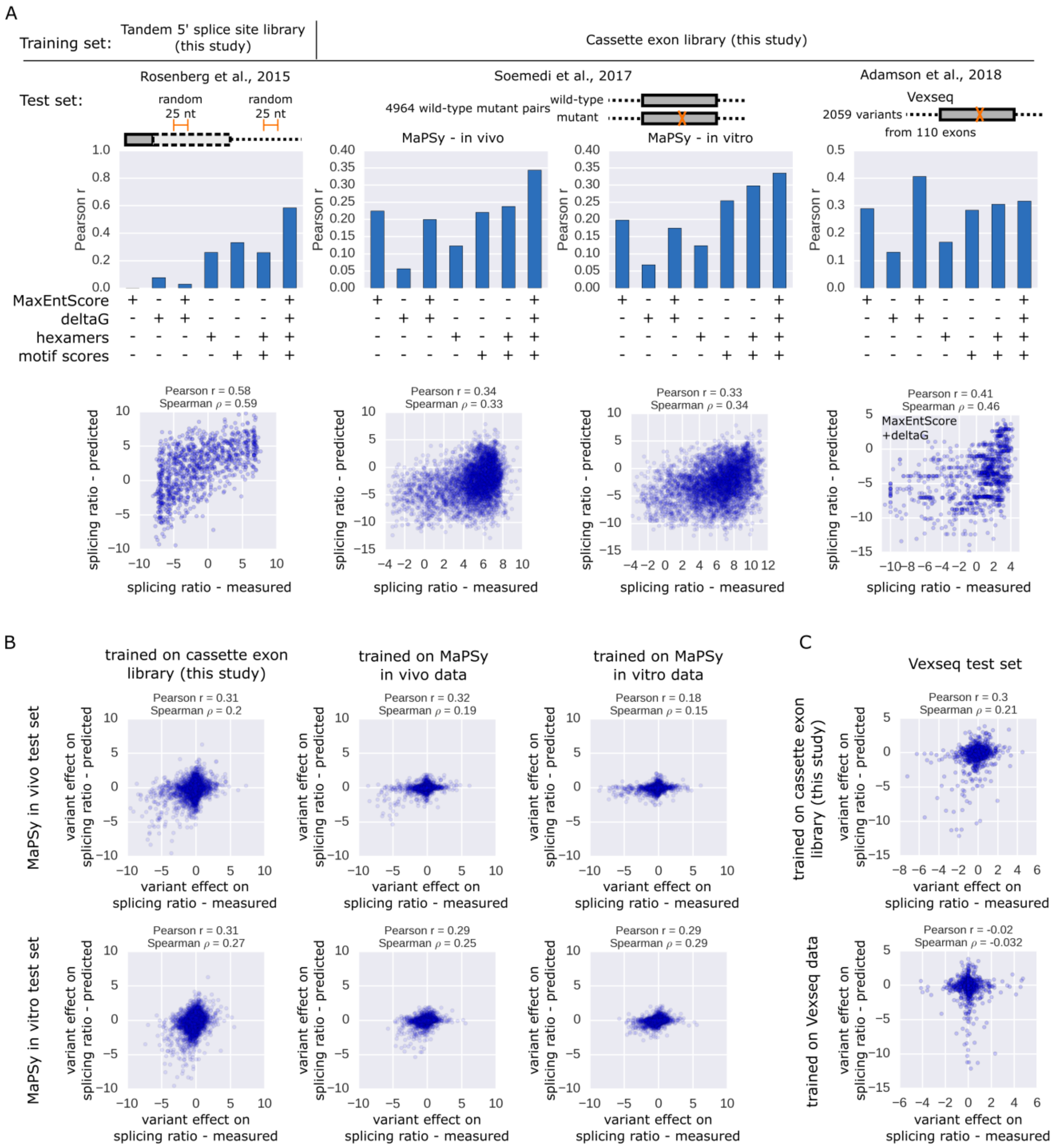

Supplementary Figure 6. Testing the model on data from other MPRAs.

A. Prediction of splicing ratios: Pearson correlation coefficients between the splicing ratio prediction of our model (trained on the data indicated above; “training set”) for sequences tested in the indicated studies (see schematic above, “test set”) and the splicing ratios measured in these studies with the indicated sets of features (top) and scatter plots of measured splicing ratios vs. predictions based on the highest scoring combination of features (bottom). BC. Prediction of variant effects based on prediction of splicing ratios for both the wild-type and the mutant sequence independently and reporting the paired difference: Scatter plots of measured variant effects vs. predictions based on the full set of features and the indicated training and test sets for data from MaPSy (B) and Vexseq (C).

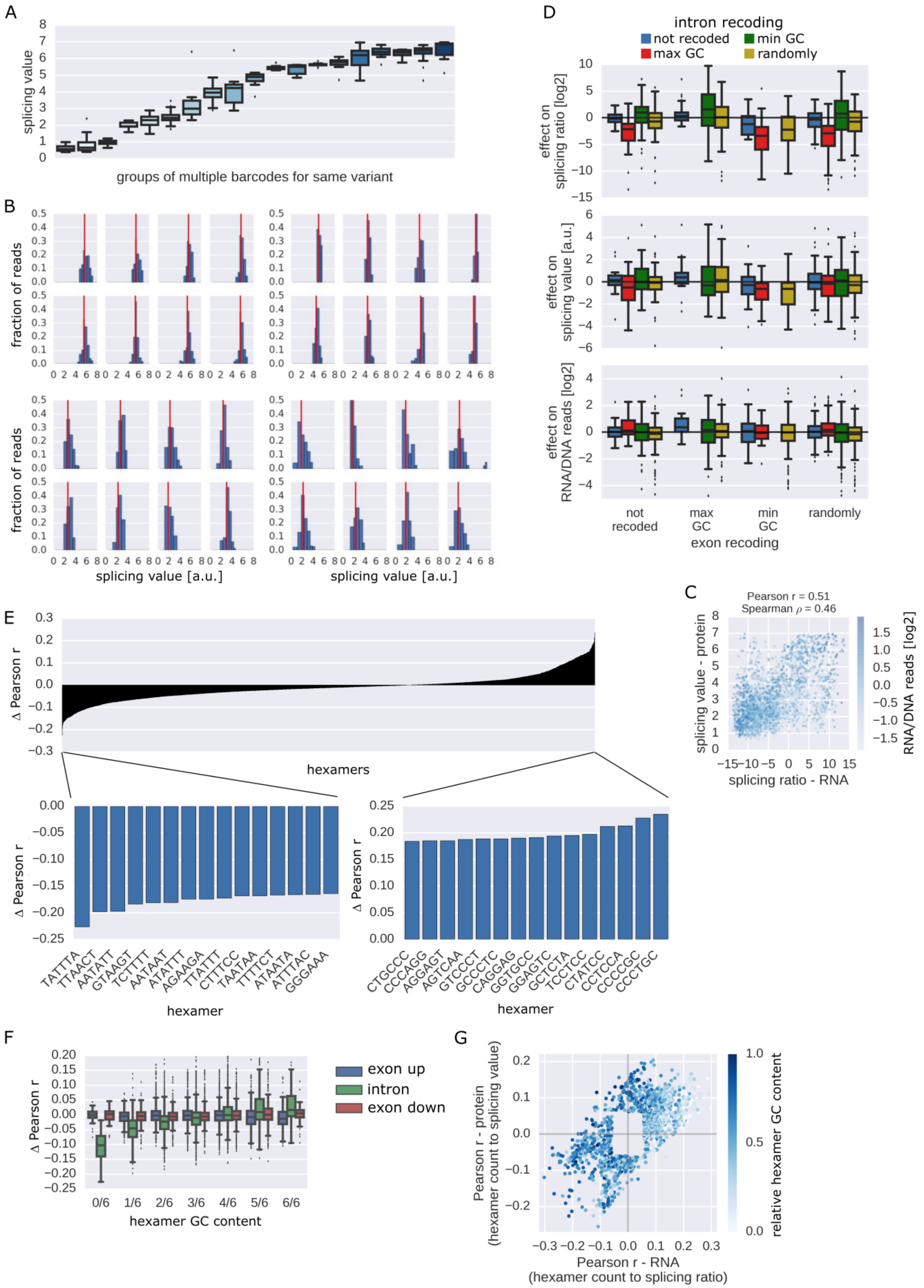
A
splicing value
groups of multiple barcodes for same variant
B
fraction of reads
splicing value [a.u.]
C
Pearson r = 0.51
Spearman ρ = 0.46
splicing value - protein
splicing ratio - RNA
RNA/DNA reads [log2]
D
intron recoding
not recoded
min GC
max GC
randomly
effect on splicing ratio [log2]
effect on splicing value [a.u.]
effect on RNA/DNA reads [log2]
not recoded
max GC
min GC
randomly
exon recoding
E
Δ Pearson r
hexamers
TATTTA TTAACT AATATT GTAAGT TCTTTT AATAAT ATATTT AGAAGA TTATTT CTTTCC TAATAA TTTTCT ATAATA ATTTAC GGGAAA
CTGCCC CCCAGG AGGAGT AGTCAA GTCCCT GCCCTC CAGGAG GGTGCC GGAGTC GCTCTA TCCTCC CTATCC CCTCCA CCCCGC CCCTGC
hexamer
F
Δ Pearson r
exon up
intron
exon down
0/6 1/6 2/6 3/6 4/6 5/6 6/6
hexamer GC content
G
Pearson r - protein
(hexamer count to splicing value)
Pearson r - RNA
(hexamer count to splicing ratio)
relative hexamer GC content

Supplementary Figure 7. A high-throughput assay for determining protein-based splicing values.

A. Boxplots showing splicing values for groups of multiple barcodes for the same variants (n>7 in each group); boxes show the quartiles of the dataset, whiskers show the range of the distribution not including outliers (displayed as points). B. Example for four barcode control groups; the distribution across bins for 8 variants with identical variable region, but different barcodes is shown. C. RNA-based splicing ratios plotted against protein-based splicing values for the tandem 5' splice sites library; the color intensity denotes the RNA expression levels (dark blue corresponds to high and light blue to low RNA expression levels (log2(RNA/DNA reads)). D. Distribution of the effect of recoding exonic and/or intronic components on RNA-based splicing ratios (top), protein-based splicing values (middle) and RNA expression levels (RNA/DNA reads; bottom) for retained introns (values normalized to the respective wild-type sequence; n=20-92 variants in each group); boxes show the quartiles of the dataset, whiskers show the range of the distribution not including outliers (displayed as points). E. Difference in Pearson correlation coefficients between all intronic hexamer counts and either RNA- or protein-based splicing readouts. F. Distribution of differences in Pearson correlation coefficients between hexamer counts in exonic and intronic regions based on the GC content of the hexamer; boxes show the quartiles of the dataset, whiskers show the range of the distribution not including outliers (displayed as points). G. Pearson correlation coefficients between intronic hexamer counts and RNA-based splicing ratios (x-axis) are plotted against Pearson correlation coefficients between the same intronic hexamer counts and protein-based splicing values. Only statistically significant correlations (after multiple testing correction, Bonferroni) are shown and color-coded for their GC content (dark blue – higher hexamer GC content; light blue – lower hexamer GC content).

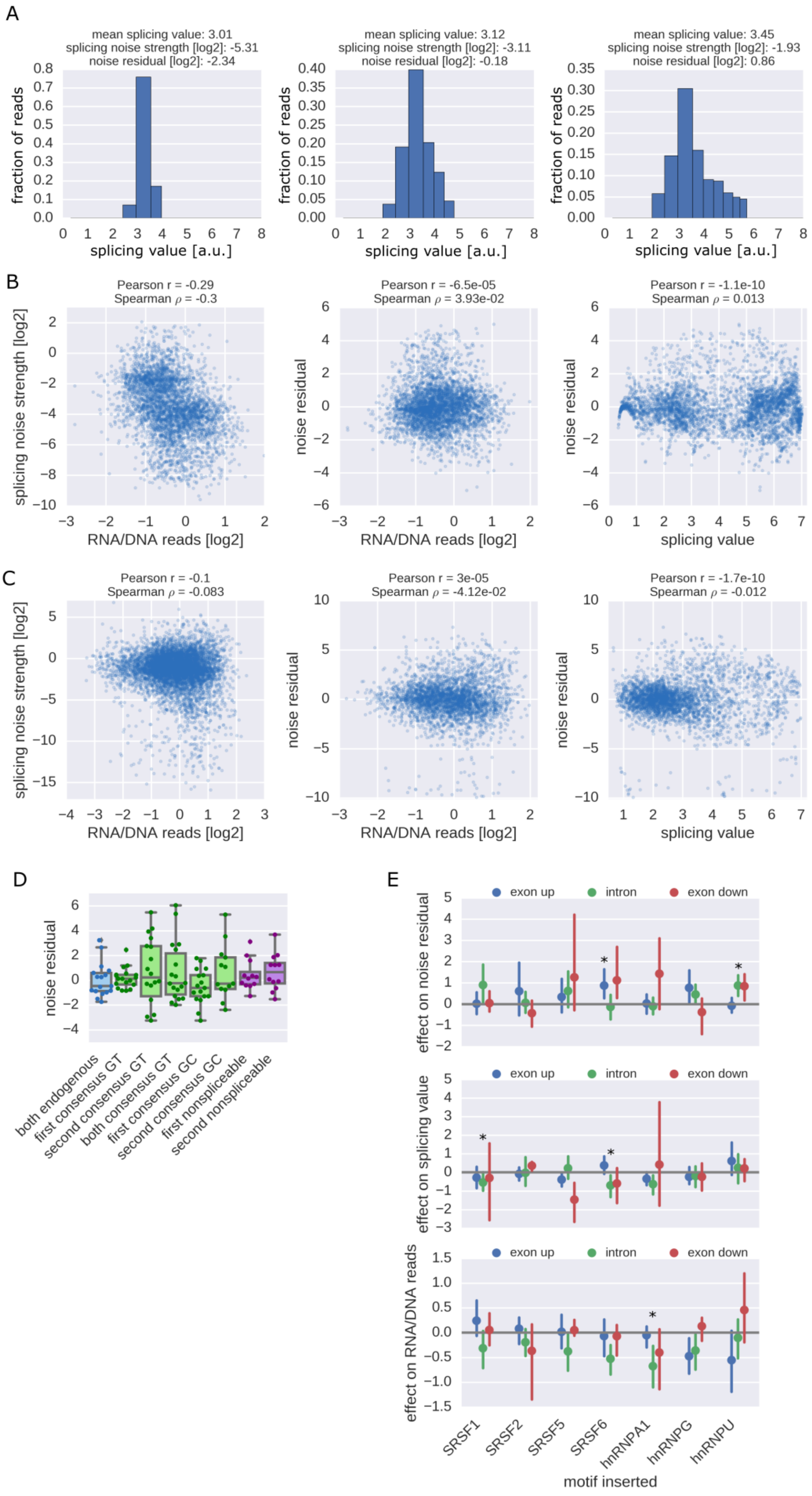
A
mean splicing value: 3.01
splicing noise strength [log2]: -5.31
noise residual [log2]: -2.34
mean splicing value: 3.12
splicing noise strength [log2]: -3.11
noise residual [log2]: -0.18
mean splicing value: 3.45
splicing noise strength [log2]: -1.93
noise residual [log2]: 0.86
fraction of reads
splicing value [a.u.]
B
Pearson r = -0.29
Spearman ρ = -0.3
Pearson r = -6.5e-05
Spearman ρ = 3.93e-02
Pearson r = -1.1e-10
Spearman ρ = 0.013
splicing noise strength [log2]
noise residual
RNA/DNA reads [log2]
splicing value
C
Pearson r = -0.1
Spearman ρ = -0.083
Pearson r = 3e-05
Spearman ρ = -4.12e-02
Pearson r = -1.7e-10
Spearman ρ = -0.012
D
noise residual
both endogenous
first consensus GT
second consensus GT
both consensus GT
first consensus GC
second consensus GC
first nonspliceable
second nonspliceable
E
exon up
intron
exon down
effect on noise residual
effect on splicing value
effect on RNA/DNA reads
SRSF1
SRSF2
SRSF5
SRSF6
hnRNPA1
hnRNPG
hnRNPU
motif inserted

Supplementary Figure 8. Assessing the regulation of splicing cell-to-cell variability.

A. Distribution across bins for three unrelated variants with similar mean splicing value, but different variance (noise). B-C. Splicing noise strength (variance/mean) for the retained introns (B) and tandem 5' splice sites (C) libraries is plotted against RNA expression (RNA/DNA reads, left). Noise residuals (after correcting for the influence of mean splicing value and expression level) are plotted against RNA expression (middle) and mean splicing value (right), for the retained introns (B) and tandem 5' splice sites (C) libraries, respectively. D. Distribution of noise residuals in variants from the tandem 5' splice sites library with the indicated sequence (endogenous, splicing consensus or nonspliceable) at the first and/or second donor splice sites; boxes show the quartiles of the dataset, whiskers show the range of the distribution not including outliers (displayed as points). E. Mean and 95% CI of the effect on noise residual (top), mean splicing value (middle) or expression level (RNA/DNA reads, bottom) of introducing a motif for the indicated splicing factor within a given region in or around a retained intron. Several points of insertion within this region are treated as one set. n=4-18 and 4-25 variants in each group).
