# Supplementary material for: Dissecting splicing decisions and cell-to-cell variability with designed sequence libraries
Source: Nat Commun. 2019 Oct 8;10:4572. doi: 10.1038/s41467-019-12642-3 (PMC6783452; doi:10.1038/s41467-019-12642-3)
Supplement: Supplementary file 4 — Description of Additional Supplementary Files [file 41467_2019_12642_MOESM4_ESM.docx]

**Description of Additional Supplementary Files**

File Name: Supplementary Data 1
Description: Native contexts used for the design of the retained intron oligonucleotide library.

File Name: Supplementary Data 2
Description: Native contexts used for the design of the cassette exon oligonucleotide library.

File Name: Supplementary Data 3
Description: Native contexts used for the design of the tandem 5’ splice sites oligonucleotide library.

File Name: Supplementary Data 4
Description: Native contexts used for the design of the tandem 3’ splice sites oligonucleotide library.

File Name: Supplementary Data 5
Description: Retained intron library, containing all the variable DNA sequences, specifications and measurements.

File Name: Supplementary Data 6
Description: Cassette exon library, containing all the variable DNA sequences, specifications and measurements.

File Name: Supplementary Data 7
Description: Tandem 5’ splice sites library, containing all the variable DNA sequences, specifications and measurements.

File Name: Supplementary Data 8
Description: Tandem 3’ splice sites library, containing all the variable DNA sequences, specifications and measurements.

File Name: Supplementary Data 9
Description: Native introns without evidence for retention in K562 (based on RNA-seq data).

File Name: Supplementary Data 10
Description: Native exons without evidence for skipping in K562 (based on RNA-seq data).

File Name: Supplementary Data 11
Description: Native donor splice sites without evidence for alternative splicing in K562 (based on RNA seq data).

File Name: Supplementary Data 12
Description: Native acceptor splice sites without evidence for alternative splicing in K562 (based on RNA seq data).

File Name: Supplementary Data 13
Description: List of all primers used in this study.

File Name: Supplementary Data 14
Description: Sequence of reporter constructs, including coordinates for library insertion sites and *mCherry* and *gfp* coding region.
