# Supplementary material for: Dissecting splicing decisions and cell-to-cell variability with designed sequence libraries
Source: Nat Commun. 2019 Oct 8;10:4572. doi: 10.1038/s41467-019-12642-3 (PMC6783452; doi:10.1038/s41467-019-12642-3)
Supplement: Supplementary file 3 — Peer Review [file 41467_2019_12642_MOESM3_ESM.pdf]

Reviewers' comments:

Reviewer #1 (Remarks to the Author):

The authors assessed the nature of splicing decisions and outcomes through a dual RNA-protein reporter assay. A library of variant sequences comprising exon-intron segments from retained introns, cassette exons and alternative 3′ and 5′ splice sites was inserted into a native genomic context in a one-sequence-per-cell manner, and the splicing outcome was determined at the RNA level by RNA-seq and at the protein level by mCherry/GFP fluorescence ratio. Through manipulation of splice site sequences, the authors reveal that when two alternative 5′ splice sites of equal strength are competing with one another typically the first one on the transcript is chosen. It is also shown that splicing outcomes can be significantly altered through other small modifications such as the insertion of splicing factor binding sites and that the effect of this change is often dependent on the previous level of splicing: strongly included exons are more likely to be disrupted through the insertion of repressor motif than exons with a lower inclusion level. Noise in splicing decisions is also examined and found to be maximal at intermediate levels of splicing. Certain sequences are tested and shown to have lower noise than would be expected by chance. The authors built predictive models for splicing outcomes and their noise which perform reasonably well.

• Figure 2G: Reason for differentiating between "exon end" and "exon"?
• Switch from 2nd/1st splice site terminology in figure 2 to adjacent/distal in figure 3. This is confusing as adjacent to the exon is 1st splice site for tandem 5′ splice sites but 2nd splice site for tandem 3′ splice sites. Recommend using 1st/2nd wording throughout.
• Relatedly, you claim that figure S3A demonstrates the ability of introns to impart their "constitutive effect" when substituted into tandem 3′ splice site contexts due to the bias in splicing towards the adjacent splice site when such a substitution is performed. Is this the splice site adjacent to the substituted intron (1st splice site) or to the exon (2nd splice site) as I would expect a shift towards the 1st splice if this was true?
• Figure S4D: Retained introns have a higher mean splicing ratio when pyrimidine tract is paired vs. unpaired indicating a shift towards the spliced product. This seems to contradict the claim of a preference for pairing in retained introns as intron retention is more likely if the tract is not paired.
• Seems more accurate to describe the "properties of optimal introns" that are less positively correlated with protein splicing value than RNA splicing ratio (Figure S5C) as being other instances of the protein-level buffering effect rather than having a "negative impact on protein-based splicing value". Are these features simply less positively correlated with splicing value than splicing ratio, or is the difference in correlation presented in the figure large enough that they actually have a negative correlation with splicing value?

Reviewer #2 (Remarks to the Author):

In "Dissecting splicing decisions and cell-to-cell variability with designed sequence libraries", Mikl et. al. assay a library of ~30k synthetic splicing reporters to investigate the effects of cis-regulatory elements on alternative splicing. The study systematically tests and confirms many previously held assumptions about alternative splicing, while offering some new insights. The study is well designed and is strengthened by providing readouts both at the RNA and protein level. The results regarding the differing effects of alternative splicing at the RNA level and protein level are intriguing and warrant future follow-up studies.
While the manuscript was generally well written, there were a few parts in the text as well as the figures that were difficult to follow. It would also be helpful if you included all legends in the figures rather than the captions. My personal preference is to be able to understand each figure can stand alone and be understood without having to necessarily refer to the text or figure caption.
Specific comments:

• In the discussion of Figure 3: "To assess the potential of individual building blocks to confer regulatory properties from one context to another, we substituted exonic and intronic components of alternative splice sites with sequences from native splice sites without evidence for alternative splicing (Fig 3A). While the constitutive effect of 3' ends of introns can generally be conferred to other contexts (Fig S3A), this is not the case for sequences surrounding the donor splice site (Fig 3B). Effects for pairs of exon and intron sequences taken from the same native context showed negative correlation (Fig 3C), hinting at antagonism between exonic and intronic components." It would be helpful if you were more explicit that you are taking exonic sequences from constitutive splicing events and placing them into the exonic positions of your synthetic splice constructs (not also testing them in intronic positions). The reference to other contexts made me initially think you were testing exonic sequences in intronic positions and vice versa. I also found the term "constitutive effect" to be confusing, although I understand it to be synonymous with splicing enhancer effect. You should elaborate on what you mean by antagonism between exonic and intronic components.
• Figure 2D would be easier to understand if you labeled the x and y axis as splicing ratios.
• On page 12, the manuscript states: "Relative intronic GC content is negatively correlated with the RNA, but not the protein splicing ratio (Fig 5C), indicating that influences of GC content on splicing efficiency are buffered at the protein level". This is interesting, but it's hard for me to imagine how intronic GC content could affect protein levels differently than RNA levels since the introns are processed in the nucleus and RNA is translated in the cytoplasm. Does relative intronic GC content take into account exonic GC content?
• On page 7, the manuscript states: "Duplicating the first or the second splice site sequence showed high correlation (Fig 2D), suggesting that the context-specific contribution to splice site choice is set by sequence properties away from the immediate splice site." A stronger statement would be that the splice site and surrounding sequence affect splicing independently of one another.

Alex Rosenberg

Reviewer #3 (Remarks to the Author):

Mikl et al. report results from an impressive large-scale reporter assay probing the effects of DNA sequence on splicing at the RNA level, protein level, as well as on cell-to-cell variability. There is a clear relevance for the readership of Nature communications because of the biological and medical relevance of splicing, and because of the several conceptual novelties that this experiment brings. However, major points must be addressed, listed below. Generally, the manuscript is too dense. While very well written, it is hard to follow. Figure legends are not explaining enough. Results are not discussed enough. The manuscript should generally discuss more what the potential mechanisms behind the reported phenomena could be, how do these findings relate to our current understanding of splicing, and what technical limitations of the experiment could alternatively explain them.

Major points
- Data. There is no supplementary table nor link to a public repository for the raw data. The current Methods description gives an idea of what has been done but neither allow reproducibility nor it allows using the data for future analysis. There are some data frames in the code folder but plain flat files should be provided and raw data shared already for revision. Supplementary tables corresponding to the different sections of the Synthetic library design Methods section are required providing the sequence of each construct, its library, its architecture (e.g.: exon, intron, splice site, GFP, mCherry coordinates). Also, a supplementary table listing the native sequences (eg: gene id, gene name, coordinate of the sequence) that have been selected should be given. This applies to "selection of suitable splice sites from publicly available RNA sequencing data for K562 cells" and to "Introns and exons of identical length with no evidence for alternative splicing in RNAseq data".

- Code. The code is shared but would not be runnable elsewhere (usage of private folders, data not

provided such as “dataframes/irdf.pkl”). Ideally one would have re-runnable code that i) fits the model from the data starting from flat files, ii) that produce the paper figures from supplementary tables. Also, the fitted model itself could be shared. A good example is the jupyter notebook that Rosenberg et al 2015 provide, which makes their resource much more useful. I understand this sets the bar high. The authors could address some of this to make their work more profitable to the community.

- “Duplicating the first or the second splice site sequence showed high correlation (Fig 2D), suggesting that the context-specific contribution to splice site choice is set by sequence properties away from the immediate splice site.”. I don’t understand this statement nor what fig2D shows. Fig 2d is not much labelled nor described. What is exactly experimentally done? How does this relate to the cartoons in 2D? What is scatter-plotted in Fig 2D?

- “Effects for pairs of exon and intron sequences taken from the same native context showed negative correlation (Fig 3C), hinting at antagonism between exonic and intronic components. “. This is very surprising and counter-intuitive. Possible mechanism and reasons should be proposed. This data actually does not require antagonism to be explained. If the strength of a site was the sum of the exonic and the intronic sequence, and if exonic and intronic sequence strengths were independently distributed, then exonic and intronic strength could be anti-correlated among the constitutive sites. It’s a so-called collider situation in causal reasoning (Berkson's paradox). The text should discuss such a possibility.

- The predictive models do not seem to exploit much of what the data analysis revealed: what about the order of the splice site, antagonism, and synergy? Could those be used in designing the model? Alternatively, are these phenomena also learned by the model? A better connection between these two parts of the paper would make sense.

- The predictive models should be compared to existing state-of-the-art ones (e.g. HAL, MMSplice) so that the added value is delineated. Doing so, one would expect a scatter-plot of the error of this model against state of the art and comment on improved predictions. Did this experimental design reveal some mechanisms that former models based on previous data could not learn?

- “recovering known splice site components in an unbiased way (Fig S4B) “ which of these hexamers are known and how (references)? Feature importance is not signed. Can we multiply it by the direction of the “average effect”. Doing so, does the direction make sense with enhancer /repressor role of the factors?

- “RNA expression levels increase with efficiency of intron removal (Fig 5CD), but protein expression levels are equally high at low and high splicing values (Fig 5F), suggesting that – on average – transcript variants lacking clear splicing signals can yield similar translational outputs as efficiently spliced variants. “

Two remarks to this. First, Does the “RNA expression levels increase” refers to the about 2-fold suggested by the moving average (Fig 5D)? Those moving averages should be provided with confidence bands. In comparison, there is also about 2-fold in Fig5E between either side of the U-shaped blue curve and the center which is not commented.

Second, the data shows that the total amount of protein (mCherry intensity) is constant while the total amount of RNA increases when the relative share of the spliced isoform (not retained intron) increases. This indicates that the spliced isoform has lower protein/mRNA ratio maybe due to lower translational efficiency or do lower protein stability than the isoform with the retained intron. This observation may be technical and related to the specific construct. The tandem 5’splice site experiment is not a good control as the protein sequence is different. The authors should be more cautious about the generality of this observation or independent experiments should be provided with an entirely different construct design. Also, what could be the mechanism? Some more discussion is

definitely needed.

- Noise: the baseline model for the noise is a linear relationship from Fig6A between splicing noise strength and splicing value. Using this baseline is not justified by some physical principle. Also, splicing value is expressed in an arbitrary unit. Panel B shows that there is still some relationship between noise residual and splicing value. Hence, considering the residual to some non-parametric fit (such as moving average, a local regression, or a GAM) would be more appropriate. Moreover, splicing noise could also be influenced by expression level. I suggest controlling for it as well when defining noise residual.

- Splicing factors reduce noise but not splicing choice. The suggestion that some splicing factors (or some splicing factor binding sites) could stabilize the decision and others enhance the choice is attractive. However, splicing factors that have been discovered so far were found because of their role in enhancing or repressing splicing. For instance, SRSF1 is known to enhance splicing. The authors should explain more how these facts can be reconciled and conduct more investigations about possible biases. These include a more careful definition of noise residual (See above). Also, the splicing ratios (i.e. splicing at the RNA level) should be checked. Another line is to compare the feature importance score between the noise model and the splicing ratio model. Altogether, the idea is interesting but this opens many questions. The tone should be more cautious.

Minor points

- Fig 1B should display what the ratio is. I guess it is always the ratio of the isoform drawn above over the isoform drawn below. Some sketched formula would make this clear.

- “This pattern held true across all potential splice site sequences (Fig S2D).” This figure has two messages: i) the overall S-shape shows that splicing is favored for the strongest of the two sites in tandem situations and ii) that for equal strength (x=0) the first is favored for 5’ but not for 3’ tandem splice sites. This figure is referred to in the main text for ii) but i) is what strikes the eye. Maybe a moving average / median together with a 95% confidence band would make ii) clearer. The dark blue vertical bars are not described and do not really help. S2D could also be referred to for i) in the main text.

- Fig S2E lacks of legend for the color scale and the numbers (I assume P-values). The P-values should be corrected for multiple testing.

- “While the constitutive effect of 3’ ends of introns can generally be conferred to other contexts”. Do you mean “transferred to other contexts”?

- “predict splicing ratios of ..” -> “predict splicing log-ratios”

- Fig 4 bottom row needs y=x diagonal line.

- 5A and text suggest that the mCherry GFP design has only been done for the intron retention cases. 5C suggests that this was also done for alternative 5’site. I lack a clear overview of the experimental design.

- 5C is for retained introns I assume. That should be labeled.

- 5D and 5E should use the same y-axis limits.

- “increased the performance of prediction to R2=0.281” it is 0.271 on Fig S6D

- In Methods, the second instance of “For prediction based on hexamers” should be “For prediction based on RBP binding sites.”

- The ATtRACT database integrates a lot of sources yielding many PWMs per factor. Also, many PWMs in ATtRACT corresponds to single gene studies with one single motif instances.
What filters have been applied?

Point-by-Point Response to the Reviewers' Comments

We would like to thank the reviewers for their insightful comments, criticism and suggestions, which – we feel – substantially improved the quality of the manuscript and greatly contributed to making our results more complete and the presentation more intuitive and intelligible. In response to the reviewers' comments, we

- added two new supplementary figures
- updated and augmented all other main and supplementary figures
- added analyses and controls to corroborate our conclusions
- added schematics and cartoons illustrating the approach throughout the manuscript in order to improve the clarity of the presentation
- made all the raw data available (NCBI GEO accession GSE132064)
- provide all the processed data (together with all the additional information requested by the reviewers) in 13 new Supplementary Tables
- completely reorganized the code and (a) made it possible to generate all the figures from Supplementary Tables and (b) shared the predictive models in a way that they can be more easily used by others and will therefore hopefully be more useful for the community (see our detailed point-by-point response to the reviewers' comments below).

Reviewer #1 (Remarks to the Author):

The authors assessed the nature of splicing decisions and outcomes through a dual RNA-protein reporter assay. A library of variant sequences comprising exon-intron segments from retained introns, cassette exons and alternative 3' and 5' splice sites was inserted into a native genomic context in a one-sequence-per-cell manner, and the splicing outcome was determined at the RNA level by RNA-seq and at the protein level by mCherry/GFP fluorescence ratio. Through manipulation of splice site sequences, the authors reveal that when two alternative 5' splice sites of equal strength are competing with one another typically the first one on the transcript is chosen. It is also shown that splicing outcomes can be significantly altered through other small modifications such as the insertion of splicing factor binding sites and that the effect of this change is often dependent on the previous level of splicing: strongly included exons are more likely to be disrupted through the insertion of repressor motif than exons with a lower inclusion level. Noise in splicing decisions is also examined and found to be maximal at intermediate levels of splicing. Certain sequences are tested and shown to have lower noise than would be expected by chance. The authors built predictive models for splicing outcomes and their noise which perform reasonably well.

• Figure 2G: Reason for differentiating between "exon end" and "exon"?

The reason behind this distinction was to create variants with different numbers of CG (or GC) dinucleotides inserted (but not varying the frequency like in the distinction between "exon (6 nt)" and "exon (9 nt)"). In revised Figure 2 (now panel H) we replaced the original labelling ("exon end") with an explicit reference to the number of insertions (3) each 6 nt.

• Switch from 2nd/1st splice site terminology in figure 2 to adjacent/distal in figure 3. This is

confusing as adjacent to the exon is 1st splice site for tandem 5′ splice sites but 2nd splice site for tandem 3′ splice sites. Recommend using 1st/2nd wording throughout.

We followed the suggestion of the reviewer and now use "splicing ratio $2^{nd}/1^{st}$ site" wording throughout (new Figure 3ABC, Figure S3ABC).

• *Relatedly, you claim that figure S3A demonstrates the ability of introns to impart their "constitutive effect" when substituted into tandem 3′ splice site contexts due to the bias in splicing towards the adjacent splice site when such a substitution is performed. Is this the splice site adjacent to the substituted intron (1st splice site) or to the exon (2nd splice site) as I would expect a shift towards the 1st splice if this was true?*

The constitutive intron is placed upstream of the first splice site. We now changed the nomenclature to "splicing ratio $2^{nd}/1^{st}$ site" following the reviewer's suggestion to keep the wording consistent throughout the manuscript. We also reorganized the presentation of the data and now present the insertion of intronic or exonic sequences in separate figures to increase the clarity. We also added schematics in Figures 3ABC and S3ABC explaining the admittedly complex design logic behind this figure.

Revised Figures 3AB and S3AB now also contain the data for wild-type library contexts without insertion of sequences from constitutive splice sites (gray data points/box plot). The potential of 3′ ends of constitutive introns to promote splicing can generally be transferred to our library contexts when inserted before the first three prime splice site, leading to low splicing ratios ($2^{nd}/1^{st}$ splice site; i.e. increased use of the 1st site) relative to the distribution for wild-type library contexts (in gray) for most 3′ ends of constitutive introns assayed (Fig S3A).

• *Figure S4D: Retained introns have a higher mean splicing ratio when pyrimidine tract is paired vs. unpaired indicating a shift towards the spliced product. This seems to contradict the claim of a preference for pairing in retained introns as intron retention is more likely if the tract is not paired.*

We agree that the original wording was confusing. By "preference for pairing in retained introns" we meant that these (potentially retained) introns have a preference for a paired pyrimidine tract in the sense that in this situation splicing of the intron is more likely. We now changed the wording to make the sentence less ambiguous (lines 223-224) and also discuss this (maybe counterintuitive) finding and the observed differences between splicing types (lines 224-231).

• *Seems more accurate to describe the "properties of optimal introns" that are less positively correlated with protein splicing value than RNA splicing ratio (Figure S5C) as being other instances of the protein-level buffering effect rather than having a "negative impact on protein-based splicing value". Are these features simply less positively correlated with splicing value than splicing ratio, or is the difference in correlation presented in the figure large enough that they actually have a negative correlation with splicing value?*

Hexamers can be both positively and negatively correlated with RNA-based splicing ratios as well as with protein-based splicing values. We agree that the difference in the Pearson correlation

coefficients shown in original Figure S5C/new Figure S7E does not provide information about the direction of the correlation, i.e. if the effects of a given hexamer on RNA and protein-based isoform ratios can be anti-correlated. We now added a scatter plot of the correlation coefficients in new Figure S7G, showing that while the majority of Hexamers being positively (or negatively) correlated with RNA-based splicing ratios show similar behavior with protein-based splicing values, a subset of hexamers do show positive correlation with one and negative correlation with the other readout.

Reviewer #2 (Remarks to the Author):

In "Dissecting splicing decisions and cell-to-cell variability with designed sequence libraries", Mikl et. al. assay a library of ~30k synthetic splicing reporters to investigate the effects of cis-regulatory elements on alternative splicing. The study systematically tests and confirms many previously held assumptions about alternative splicing, while offering some new insights. The study is well designed and is strengthened by providing readouts both at the RNA and protein level. The results regarding the differing effects of alternative splicing at the RNA level and protein level are intriguing and warrant future follow-up studies.

While the manuscript was generally well written, there were a few parts in the text as well as the figures that were difficult to follow. It would also be helpful if you included all legends in the figures rather than the captions. My personal preference is to be able to understand each figure can stand alone and be understood without having to necessarily refer to the text or figure caption.

We followed the reviewer's recommendation and tried to make the figures more self-explanatory by adding legends directly onto the figures and adding schematics, cartoons and information to make the at times complex design logic more intelligible. Specifically this was done for Figs 1B, 2AD, 3ABCD, 5ABH, S1A, S2ABC, S2D, S3AB, S4C, S4BCD, S6A, S7CG, S8ABC.

Specific comments:

• In the discussion of Figure 3: "To assess the potential of individual building blocks to confer regulatory properties from one context to another, we substituted exonic and intronic components of alternative splice sites with sequences from native splice sites without evidence for alternative splicing (Fig 3A). While the constitutive effect of 3' ends of introns can generally be conferred to other contexts (Fig S3A), this is not the case for sequences surrounding the donor splice site (Fig 3B). Effects for pairs of exon and intron sequences taken from the same native context showed negative correlation (Fig 3C), hinting at antagonism between exonic and intronic components." It would be helpful if you were more explicit that you are taking exonic sequences from constitutive splicing events and placing them into the exonic positions of your synthetic splice constructs (not also testing them in intronic positions). The reference to other contexts made me initially think you were testing exonic sequences in intronic positions and vice versa. I also found the term "constitutive effect" to be confusing, although I understand it to be synonymous with splicing enhancer effect. You should elaborate on what you mean by antagonism between exonic and intronic components.

In the revised manuscript we reorganized the presentation of the data and now present the insertion of intronic or exonic sequences in separate figures to increase the clarity. We also added

schematics in Figures 3ABC and S3ABC explaining the admittedly complex design logic behind this figure. In the text we tried to be more explicit and consistent and considerably extended the discussion of this figure, including a hopefully clearer graphical (new Figure 3C, S3C, 3D) and textual (lines 155-183; as well as lines 198-208 for the case of cassette exons) explanation of what we mean by antagonism between exonic and intronic components. Specifically we also rephrased the sentence containing the term "constitutive effect of 3' ends of introns" and refer to it now as "the potential of 3' ends of constitutive introns to promote splicing".

• *Figure 2D would be easier to understand if you labeled the x and y axis as splicing ratios.*

We changed the axis labels accordingly (now this is panel E in Figure 2), added information in the schematic above and tried to connect the axis labels with the color scheme used in the legend. We also explain and discuss this Figure more extensively in the revised manuscript (lines 114-120).

• *On page 12, the manuscript states: "Relative intronic GC content is negatively correlated with the RNA, but not the protein splicing ratio (Fig 5C), indicating that influences of GC content on splicing efficiency are buffered at the protein level". This is interesting, but it's hard for me to imagine how intronic GC content could affect protein levels differently than RNA levels since the introns are processed in the nucleus and RNA is translated in the cytoplasm. Does relative intronic GC content take into account exonic GC content?*

Yes, relative intronic GC takes into account exonic GC. Absolute intronic GC content shows similar results, and we now report correlation coefficients for both in revised Figure 5C. We agree that it would be hard to imagine how the GC content of an already removed intron could affect downstream processing. We therefore suggest (and have added this in the revised manuscript, lines 308-313) that the buffering happens through the unspliced isoform. High GC content could lead to increased nuclear retention as a result of initiated, but ultimately unsuccessful removal of the intron. Alternatively, one could imagine a mechanism by which higher GC content could lead to reduced translation (maybe due to more rigid secondary structure, etc).

• *On page 7, the manuscript states: "Duplicating the first or the second splice site sequence showed high correlation (Fig 2D), suggesting that the context-specific contribution to splice site choice is set by sequence properties away from the immediate splice site." A stronger statement would be that the splice site and surrounding sequence affect splicing independently of one another.*

We thank the reviewer for the suggestion. We agree and added this conclusion in the revised manuscript as part of a more extensive discussion of this Figure (now Fig 2E; lines 114-120).

Alex Rosenberg

Reviewer #3 (Remarks to the Author):

Mikl et al. report results from an impressive large-scale reporter assay probing the effects of DNA sequence on splicing at the RNA level, protein level, as well as on cell-to-cell variability. There is a clear relevance for the readership of Nature communications because of the biological and medical relevance of splicing, and because of the several conceptual novelties that this experiment brings. However, major points must be addressed, listed below. Generally, the manuscript is too dense. While very well written, it is hard to follow. Figure legends are not explaining enough. Results are not discussed enough. The manuscript should generally discuss more what the potential mechanisms behind the reported phenomena could be, how do these findings relate to our current understanding of splicing, and what technical limitations of the experiment could alternatively explain them.

We tried to make the figures more self-explanatory – as also suggested by Reviewer 2 – by adding legends directly onto the figures and adding schematics, cartoons and information to make the at times complex design logic more intelligible. Specifically this was done for Figures 1B, 2AD, 3ABCD, 5ABH, S1A, S2ABC, S2D, S3AB, S4C, S4BCD, S6A, S7CG, S8ABC. In addition, we also expanded the figure legends, specifically for Figures 1, 3, 5 and 6 and Supplementary Figures S3, S5, S6, S7 and S8.

We significantly expanded the explanation and discussion of particularly complex parts of the manuscript, e.g. lines 110-120 in the discussion of Figure 2E and S2D, lines 155-208 in the discussion of Figure 3 and lines 223-274 and 404-413 in the discussion of our model (Fig 4, Fig S4 and new Figures S5 and S6).

Following the reviewer's suggestions we now discuss potential mechanisms and explanations for our observations as well as potential technical limitations in greater depth, for example in the discussion of the "antagonism" between intronic and exonic sequences (specifically lines 173-178 and 201-208), a potential dichotomy between constitutive and alternative splice sites (lines 189-193), the preference for the pyrimidine tract to be paired in the removal of (potentially retained) introns (lines 223-231), the interpretation of binding site motifs (lines 251-255), the applicability of our model on other datasets and contexts (liens 256-274 and 404-413), the discrepancies between RNA and protein isoform ratios (lines 297-300, 308-313), the interaction of mean splicing value and noise (lines 365-369) and the effect of splice site sequence on splicing noise residual (lines 374-376).

Major points

- Data. There is no supplementary table nor link to a public repository for the raw data. The current Methods description gives an idea of what has been done but neither allow reproducibility nor it allows using the data for future analysis. There are some data frames in the code folder but plain flat files should be provided and raw data shared already for revision. Supplementary tables corresponding to the different sections of the Synthetic library design Methods section are required providing the sequence of each construct, its library, its architecture (e.g.: exon, intron, splice site, GFP, mCherry coordinates). Also, a supplementary table listing the native sequences (eg: gene id, gene name, coordinate of the sequence) that have been selected should be given. This applies to "selection of suitable splice sites from publicly available RNA sequencing data for K562 cells" and to "Introns and exons of identical length with no evidence for alternative splicing in RNAseq data".

Raw data have now been uploaded to the NCBI GEO and are available under accession GSE132064.

All the requested information has now been made available in 13 new supplementary tables: Tables S1-S4 contain information on the native sequence contexts that were used as starting points for the rational design for each of the four splicing types assayed here. Tables S5-S8 contain all the processed data extracted from the raw data and information on all >32,000 library variants. The different synthetic library design subsets are marked and this subdivision is also referred to in the revised methods section (lines 480, 487, 494, 519, 527, 535 and 541-542). Tables S9-S12 contain information on the native introns and exons with no evidence for alternative splicing in RNAseq data for the four splicing types. Table S13 contains information on the reporter constructs, including the full sequence of the transcribed region and coordinates for GFP, mCherry, library insertion sites and additional splice sites.

In the completely revised code provided to the reviewers we now base all design and analysis steps on the new supplementary tables provided. Upon acceptance the code will be shared in a public repository.

- *Code. The code is shared but would not be runnable elsewhere (usage of private folders, data not provided such as "dataframes/irdf.pkl"). Ideally one would have re-runnable code that i) fits the model from the data starting from flat files, ii) that produce the paper figures from supplementary tables. Also, the fitted model itself could be shared. A good example is the jupyter notebook that Rosenberg et al 2015 provide, which makes their resource much more useful. I understand this sets the bar high. The authors could address some of this to make their work more profitable to the community.*

We reorganized the code following the reviewer's suggestions. Python files generating the designed sequence libraries are shared and start from the supplementary files containing the native contexts (Tables S1-S4, S9-S12). The raw data is shared (see above) and the mapping and processing can be re-run with the provided code. All the figures can now be generated from the supplementary tables containing the processed data (Tables S5-S8; Python files named Figure1 through 6). The training and testing of the models is kept separate as this might be the part of the code most likely to be used by others. All the models as well as training and test sets (which in addition can also be generated from the supplementary files) are shared, but can in addition also be generated from the supplementary tables and the provided code.

To maximize usability we adapted the code in a way that a potential user only has to provide a csv file with a primary sequence (of any (reasonable) length; it can be expected that the model performs best with exon/intron lengths that are close to the ones of the training set) and coordinates of exon/intron boundaries. Apart from all the python files generating the figures and training and testing the model, we now also provide a way to easily get predictions for any set of input sequences (run_predictor.py in the code provided with this manuscript, including test input and output for each of the four splicing types; see the Readme file for details). Upon acceptance the code will be shared in a public repository.

- "Duplicating the first or the second splice site sequence showed high correlation (Fig 2D), suggesting that the context-specific contribution to splice site choice is set by sequence properties away from the immediate splice site.". I don't understand this statement nor what fig2D shows. Fig 2d is not much labelled nor described. What is exactly experimentally done? How does this relate to the cartoons in 2D? What is scatter-plotted in Fig 2D?

We apologize for the insufficient explanation and discussion of this figure. In the revised manuscript we aimed to improve the presentation both in the figure itself (which is now panel E in Figure 2) and in the results section. We expanded the discussion in the main text substantially, and the sentence quoted in the reviewer's comment was replaced with the following paragraph:

"Artificially creating a situation of equal splice site strength by copying the first (orange) splice site sequence to the second donor showed high correlation (Fig 2E) with the inverse configuration (second (green) splice site sequence copied to the first donor), suggesting that the immediate splice site sequence and the larger sequence context affect splicing independently from one another. Situations where the "first come-first served" rule is broken (positive ratios of 2nd/1st splice site usage) are therefore mostly set by sequence properties away from the immediate splice site." (lines 114-120)

We also changed the axis labels according to a comment from reviewer 2 (now this is panel E in Figure 2), added information in the schematic above and tried to connect the axis labels with the color scheme used in the legend. We hope that all of these changes together make this result more accessible for the reader.

- "Effects for pairs of exon and intron sequences taken from the same native context showed negative correlation (Fig 3C), hinting at antagonism between exonic and intronic components. ". This is very surprising and counter-intuitive. Possible mechanism and reasons should be proposed. This data actually does not require antagonism to be explained. If the strength of a site was the sum of the exonic and the intronic sequence, and if exonic and intronic sequence strengths were independently distributed, then exonic and intronic strength could be anti-correlated among the constitutive sites. It's a so-called collider situation in causal reasoning (Berkson's paradox). The text should discuss such a possibility.

In tandem 5' splice sites we observe a difference in the distribution between variants containing a constitutive exon (splicing ratio $1^{st}/2^{nd}$ = adjacent/distal: 3.1+/-7.3 (mean+/-standard deviation)) and those containing a constitutive intron (splicing ratio $2^{nd}/1^{st}$ = adjacent/distal: 0.5+/-7.0 (mean+/-standard deviation)). This difference is significant (p=0.004, Wilcoxon signed rank test) and shows that the exonic components have a slightly stronger effect on splice site choice. We cannot definitively exclude the possibility that this gives rise to the anticorrelation (which in revised Figure 3C appears as positive correlation, as we now stick to the wording splicing ratio $2^{nd}/1^{st}$ throughout the manuscript, as suggested by the other reviewers). However, (a) the large overlap between the distributions, (b) the observation that individual sequences behave similar in all the library contexts (e.g. both parts of constitutive site 8667 promoting splicing at the adjacent site, while e.g. for 21693 only the intron has a strong positive influence on splicing at the upstream donor site) and (c) the fact that in the case of tandem 3' splice sites the difference is much more pronounced (7.1+/-5.2 vs 0.1+/-4.3; $p=1.1*10^{-29}$), yet no (anti) correlation can be observed) lead us to believe that we

observe actual antagonism between exonic and intronic elements here. We also mention this in the main text and added a significant amount of discussion for this figure (lines 155-193) and use more careful wording when referring to the proposed antagonism between components of native constitutive donor sites (e.g. "…suggesting that 'antagonistic' behavior of exonic and intronic components…", line 172-173).

We also extended this analysis to the other libraries (Fig S3F-I, lines 201-208) and found that the anticorrelation between the effect of exons and their surrounding introns cannot be explained by differences between the groups as a whole (whereas for retained introns we do find differences between the groups, but no or a much weaker negative correlation). All of this leads us to assume, that the effect indeed constitutes an examples of antagonism.

We now added a discussion of potential mechanisms and reasons in the revised manuscript, for both tandem 5' splice sites (lines 173-178) and cassette exons (lines 206-208). Briefly, we speculate that in the native situation and in the absence of a competing donor site, strong specification of a 5' splice site on either the exonic and intronic side is enough to ensure efficient usage of this splice site, avoiding redundancy and potentially also allowing for evolutionary plasticity. In the case of cassette exons, antagonism between splicing enhancers and repressors has been described in the past (e.g. Hua et al., 2008, AJHG; 10.1016/j.ajhg.2008.01.014). Here we now provide evidence that this design principle also exists at the level of building blocks (exons, introns) as a whole.

- The predictive models do not seem to exploit much of what the data analysis revealed: what about the order of the splice site, antagonism, and synergy? Could those be used in designing the model? Alternatively, are these phenomena also learned by the model? A better connection between these two parts of the paper would make sense.

The results of the data analysis influenced the model in terms of the sets of features used and the type of approach we took. We chose to use Gradient Boosting Trees as a prediction algorithm as they are able to capture non-linear interactions between features, i.e. antagonism and coordination or location-dependent effects. One example for conclusions drawn from model interpretations that match the results of the data analysis is the dominance of the first of tandem 5' splice sites, which is reflected in the impact the features have on the model, in particular secondary structure around the splice site. This is now included in the revised manuscript in Figure S4D and discussed in lines 232-235.

- The predictive models should be compared to existing state-of-the-art ones (e.g. HAL, MMSplice) so that the added value is delineated. Doing so, one would expect a scatter-plot of the error of this model against state of the art and comment on improved predictions. Did this experimental design reveal some mechanisms that former models based on previous data could not learn?

We do not claim to provide a predictor that will work equally well on random input sequences. Our goal rather is to show the level of prediction accuracy that can be achieved when controlling for many additional factors like the genomic environment, larger sequence context, promoter strength etc. In the revised manuscript, we now test our predictor on other datasets (new Fig S6; lines 256-274; in particular MaPSy and Vexseq datasets and data from Rosenberg et al., 2015, and perform reasonably well (new Fig S6, Pearson r between 0.33 and 0.58), both predicting splicing ratios from

primary sequence alone and the effect of sequence mutations. We want to point out that our model is neither trained nor designed for prediction of variant effects (the prediction goal was to predict log-ratios of two alternative splicing options based on primary sequence alone). Controlling the genomic and local context to such a large degree allows us to achieve high prediction scores, but also limits the generalizability of the model. This is now extensively discussed both in the results as well as the discussion section of the revised manuscript (lines 256-274 and 404-413).

In terms of new insights into mechanisms derived from our model we show - apart from now explicitly addressing the sensitivity of the predictive power to the genomic context and experimental approach in the manuscript – that very simple secondary structure features alone, without any primary sequence information, are predictive of splicing behavior (Fig 4), and that this also holds true for prediction of splicing behavior in other contexts and measured in other experimental frameworks (new Fig S6).

- *"recovering known splice site components in an unbiased way (Fig S4B) " which of these hexamers are known and how (references)? Feature importance is not signed. Can we multiply it by the direction of the "average effect". Doing so, does the direction make sense with enhancer /repressor role of the factors?*

In this case we were referring to hexamers that correspond to canonical donor and acceptor sites (eg. GUAAGU and UUUCAG). We now point this out more explicitly in the revised manuscript (lines 238-243). We replaced the original plots showing feature importance with SHAP plots (http://arxiv.org/abs/1802.03888), which contain much more information, in particular also about the direction in which a specific feature influences the prediction. We also discuss this in the revised manuscript (lines 236-255) and explicitly refer to the position-dependent effect of a hexamer feature constituting a canonical donor site on prediction of splicing ratios of tandem 5' splice sites (Fig S4E, lines 241-243) and the positive effect of members of the SR protein family of splicing factors on exon inclusion (Fig S5, lines 251-255).

- *"RNA expression levels increase with efficiency of intron removal (Fig 5CD), but protein expression levels are equally high at low and high splicing values (Fig 5F), suggesting that – on average – transcript variants lacking clear splicing signals can yield similar translational outputs as efficiently spliced variants. "*

Two remarks to this. First, Does the "RNA expression levels increase" refers to the about 2-fold suggested by the moving average (Fig 5D)? Those moving averages should be provided with confidence bands. In comparison, there is also about 2-fold in Fig5E between either side of the U-shaped blue curve and the center which is not commented.

We now provide the moving averages with 95% confidence intervals (as determined using bootstrapping) in new Figure 5DE. We also discuss the drop in RNA and protein expression levels in Figures 5EFG (lines 302-305), which we attribute to a higher chance for failed processing and subsequently degradation associated with a situation where there is not one dominant splicing choice (i.e. intermediate splicing levels).

Second, the data shows that the total amount of protein (mCherry intensity) is constant while the total amount of RNA increases when the relative share of the spliced isoform (not retained intron) increases. This indicates that the spliced isoform has lower protein/mRNA ratio maybe due to lower translational efficiency or do lower protein stability than the isoform with the retained intron. This observation may be technical and related to the specific construct. The tandem 5'splice site experiment is not a good control as the protein sequence is different. The authors should be more cautious about the generality of this observation or independent experiments should be provided with an entirely different construct design. Also, what could be the mechanism? Some more discussion is definitely needed.

The total amount of protein does not increase overall, but it does show the characteristic dip at intermediate splicing values observed also for tandem 5' splice sites at both the RNA and the protein level, which we attribute to a higher chance for failed processing and degradation in situations of less clear-cut splicing decisions. In our view the surprising aspect is that the protein expression level is equally high at low splicing values. As an explanation for this phenomenon we now propose that it might be due to the fact that variants with such sub-optimal introns are not even recognized as a potential target for the splicing machinery, which might allow them to avoid any delay or retention associated with inefficient processing by the spliceosome, leading to higher translational output (lines 298-300).

Regarding the difference in protein sequence, we believe that the data for the retained intron library can be compared to the tandem 5' splice site library as the retained intron library consists of variants with completely different protein sequences (based on 38 native contexts) and the only sequences in common (the upstream mCherry and the downstream GFP) are also present in the tandem 5' library. We do however agree that this does not constitute an adequate experimental control due to the inherent difference in the splicing decision (to splice or not to splice in the case of retained introns vs. the choice between two donors in the case of tandem 5' splice sites) and refer to this difference in the manuscript (lines 300-302). We now state explicitly that these conclusions probably are specific to the particular context of a gene with a single intron (line 297).

- Noise: the baseline model for the noise is a linear relationship from Fig6A between splicing noise strength and splicing value. Using this baseline is not justified by some physical principle. Also, splicing value is expressed in an arbitrary unit. Panel B shows that there is still some relationship between noise residual and splicing value. Hence, considering the residual to some non-parametric fit (such as moving average, a local regression, or a GAM) would be more appropriate. Moreover, splicing noise could also be influenced by expression level. I suggest controlling for it as well when defining noise residual.

We agree with the reviewer that there is a non-linear relationship between noise and mean splicing value. In order to avoid effects of the mean splicing value on our noise analyses as much as possible we follow the reviewer's suggestion and now fit a generalized additive model to control for this dependence. Noise strength is correlated with expression levels (new Fig S8B), but this appears to be a consequence of the correlation between expression level and mean splicing value as controlling for the mean splicing value alone also abolishes the association between expression level and noise strength to a large extent (data not shown). We nevertheless included expression

level in the generalized additive model (as a linear term; lines 350-354, lines 771-774) and explicitly also control for it, leading to a lack of correlation between noise residual and both mean splicing value and expression level (Fig S8BC).

We repeated all analyses of the effect of sequence changes on noise residual and observed an increase in noise residual when a donor site with an intron initial GC was introduced. In our view this seems intuitive given that it is the suboptimal variant for the major spliceosome (as opposed to intron initial GT) which might result in larger uncertainty in processing. We also extended our noise analysis to tandem 5' splice sites and saw that for a subset of retained introns the cell-to-cell variability is "encoded" in the sequence, this is not or only rarely the case for tandem 5' splice sites (new Fig 6D, bottom; lines 359-363), suggesting differences in the extent to which the cell-to-cell variability of a splicing decision is controlled depending on the splicing type.

- Splicing factors reduce noise but not splicing choice. The suggestion that some splicing factors (or some splicing factor binding sites) could stabilize the decision and others enhance the choice is attractive. However, splicing factors that have been discovered so far were found because of their role in enhancing or repressing splicing. For instance, SRSF1 is known to enhance splicing. The authors should explain more how these facts can be reconciled and conduct more investigations about possible biases. These include a more careful definition of noise residual (See above). Also, the splicing ratios (i.e. splicing at the RNA level) should be checked. Another line is to compare the feature importance score between the noise model and the splicing ratio model. Altogether, the idea is interesting but this opens many questions. The tone should be more cautious.

Controlling for non-linear associations between noise strength and mean splicing value and also for an effect of expression level significantly changed our conclusions about the effect of splicing factor binding sites on noise residual (revised Fig 6H, S8E; lines 378-382). We think that some of the initial observations (when we did not control for non-linear associations between mean and noise) might have been a consequence of a (minor) effect on mean splicing values. We changed this part of the manuscript accordingly and toned down our conclusion. The general message that splicing factor binding sites have the potential to affect noise residuals independently, however, still holds true even after carefully controlling for residual associations. We now also identified an increase in noise residual in variants with an intron initial GC (new Fig 6G, lines 370-378), providing another example where splice site properties affect splicing noise.

Minor points

- Fig 1B should display what the ratio is. I guess it is always the ratio of the isoform drawn above over the isoform drawn below. Some sketched formula would make this clear.

We added this information also in the axis label of the histograms in revised Fig 1B.

- "This pattern held true across all potential splice site sequences (Fig S2D)." This figure has two messages: i) the overall S-shape shows that splicing is favored for the strongest of the two sites in tandem situations and ii) that for equal strength (x=0) the first is favored for 5' but not for 3' tandem splice sites. This figure is referred to in the main text for ii) but i) is what strikes the eye. Maybe a moving average / median together with a 95% confidence band would make ii) clearer. The dark blue vertical bars are not described and do not really help. S2D could also be referred to for i) in the main text.

We agree with the reviewer and added a moving average with 95% confidence intervals (as determined by bootstrapping; revised Figure S2D). We also comment on the sigmoid shape of the relationship as being an indication for the dominance of one site in the situation of different splice site strengths (line 112-114) and thank the reviewer for sharing this observation.

- Fig S2E lacks of legend for the color scale and the numbers (I assume P-values). The P-values should be corrected for multiple testing.

This has been corrected now. Revised Fig S2E reports corrected P-values (by splicing type) and the missing legend has been added.

- "While the constitutive effect of 3' ends of introns can generally be conferred to other contexts". Do you mean "transferred to other contexts"?

Yes. To improve the clarity we changed this sentence to "The potential of 3' ends of constitutive introns to promote splicing can generally be transferred to our library contexts, leading to low splicing ratios" (lines 179-180).

- "predict splicing ratios of .." -> "predict splicing log-ratios"

This has been changed (lines 249-250).

- Fig 4 bottom row needs y=x diagonal line.

This has been added (revised Fig 4).

- 5A and text suggest that the mCherry GFP design has only been done for the intron retention cases. 5C suggests that this was also done for alternative 5'site. I lack a clear overview of the experimental design.

We now added the experimental overview also for the alternative 5' splice site library (Fig 5A) and explain the design in the text (lines 280-281).

- 5C is for retained introns I assume. That should be labeled.

The label has been added.

- 5D and 5E should use the same y-axis limits.

This has been changed in revised Figure 5D and 5E.

- "increased the performance of prediction to R2=0.281" it is 0.271 on Fig S6D

The values for the prediction of a noise residual corrected for non-linear interactions with mean splicing values changed, also due to the fact that the mean splicing value by itself is now not predictive anymore (revised Fig 6E).

- In Methods, the second instance of "For prediction based on hexamers" should be "For prediction based on RBP binding sites."

This has been changed.

- The ATtRACT database integrates a lot of sources yielding many PWMs per factor. Also, many PWMs in ATtRACT corresponds to single gene studies with one single motif instances. What filters have been applied?

We filtered the ATtRACT database for human motifs, all further selection was based on the "usefulness" for the prediction (results lines 244-248, methods lines 798-800). We did not want to exclude e.g. RBPs that (so far) have not been implicated in splicing as we (a) cannot rule out that they actually do influence splicing and (b) in the case of protein splicing values and noise residual we do use readouts that will probably be affected by factors not directly acting on the splicing reaction itself, but might differentially affect splice isoforms. We agree that some PWMs in the database are more reliable and informative than others, but since we in any case do not demonstrate that these motifs are actually bound, we refer to them as "binding sites" unless we explicitly speculate about a biological explanation for an observed phenomenon.

REVIEWERS' COMMENTS:

Reviewer #2 (Remarks to the Author):

The authors have put substantial effort into addressing my concerns as well as the concerns of the other reviewers. I specifically had some concerns about clarity that the authors have now addressed. The work is impressive and should be of interest to the wider splicing community as well as others working on functional genomics. I recommend that the paper be published in Nature Communications.

Reviewer #3 (Remarks to the Author):

Overall the authors did a remarkable work to address my points and those of the other reviewers. The manuscript should be published now.

Julien Gagneur
